# Adsorption of Acid Yellow 36 and direct blue 86 dyes to *Delonix regia* biochar-sulphur

**DOI:** 10.1038/s41598-025-85405-4

**Published:** 2025-01-27

**Authors:** Ahmed Eleryan, Uyiosa Osagie Aigbe, Kingsley Eghonghon Ukhurebor, Mohamed A. Hassaan, Safaa Ragab, Otolorin Adelaja Osibote, Ismail Hossain, Ahmed El Nemr

**Affiliations:** 1 National Institute of Oceanography and Fisheries (NIOF), Kayet Bey, Elanfoushy, Alexandria, Egypt; 2 Department of Mathematics and Physics, Cape Peninsula University of Technology, Cape Town, South Africa; 3 Department of Physics, Edo State University, Uzairue, Edo State Nigeria; 4 Department of Nuclear and Renewable Energy, Ural Federal University, Yekaterinburg, Russia

**Keywords:** Adsorption, Biochar, Dye removal, Delonix regia seed pods, Wastewater treatment, Environmental chemistry, Chemical engineering

## Abstract

This study aims to investigate a new approach to removing hazardous dyes like Direct Blue 86 (DB86) and Acid Yellow 36 (AY36) from aqueous environments. *Delonix regia* biochar-sulphur (DRB-S), made from *Delonix regia* seed pods (DPSPs), is an inexpensive and environmentally friendly adsorbent. Different characterization investigations using BJH, BET, FTIR, SEM, DSC, TGA, and EDX were utilized in the descriptions of the DRB-S biosorbent. The optimal pH for AY36 dye and DB86 dye adsorption to the DRB-S adsorvbent was at pH 1.5. For the adsorption of AY36 and DB86 to DRB-S, equilibrium was attained at 30 and 90 min of reaction time interaction. The Langmuir model (LGM) and pseudo-second-order-model (PSOM) best describe the biosorption of both dye molecules to the biosorbent owing to the equal and homogeneous spread of the dye molecules over the biosorbent porous surface and a chemisorption process which involved the valency force through the exchange of electrons between the dye molecules and the prepared biosorbent. The determined biosorption capacities for both dyes (AY36 and DB86) were found to be 270.27 mg/g and 36.23 mg/g, respectively. In conclusion, this recently synthesised DRB-S adsorbent exhibited an impressive sorption capacity and successfully removed AY36 and DB86 dyes. This suggests that the biosorbent has potential applications in wastewater treatment and can be recycled without affecting its adsorption effectiveness.

**Supplementary Information:**

The online version contains supplementary material available at 10.1038/s41598-025-85405-4.

## Introduction

Globally, a serious environmental issue is water contamination. Numerous toxins, mostly released during industrial and agricultural processes and some domestic activities, substantially impact water contamination^1–4^. Discharging untreated dye effluents is one of the main causes of water pollution. These dyes are toxic to humans as well as plants and aquatic life. Consequently, one of the most prevalent contaminants in freshwater is dyes, released by various industrial sectors, including textiles, paint, leather, cosmetics, paper, and pulp. Even in deficient concentrations, dyes can aesthetically impact water bodies^5,6^. Non-biodegradable dyes harm terrestrial and atmospheric ecosystems when discharged or used in other ways, harming aquatic ecosystems by lowering the amounts of dissolved oxygen in water bodies^7,8^.

According to their chemical structure, especially chromophore moieties responsible for colour, dyes used in textiles are considered into different classess as azo, triphenylmethane, anthraquinone, cyanine, indigoid, xanthene, etc., with azo dyes being one of the largest classes of commercialized synthetic dyes^9–11^. Acid Yellow 36 (AY36) also known as Metanil Yellow is a poisonous azo dye with chemical formula C_18_H_14_N_3_NaO_3_S and molecular weight of 375.4 g/mol. Owing to it being water soluble, it is used leather, paper, beverage and textile industries. According to animal research, it is hepatoxic and neurotoxic. When it comes in contact with the skin, it induces allergic dermatitis and noxious methaemoglobinaemia and cyanosis in humans. It is indicative this dye shows tumour producing effects and may lead to intestinal and enzymic disorder in the body of humans^12^. Direct blue 86 (DB86), also known as Direct Fast Blue GL or Direct Fast Turquoise Blue GL with a chemical formula C_32_H_14_CuN_8_Na_2_O_6_S_2_, is a grey blue to blue powder, well soluble in water and an anionic commercial dye generally applied in paper industries, viscose dyeing and printing, wool and organic pigments manufacturing and cotton printing^13,14^. For colouring fibres such as wool, silk, and other textiles, typical azo dyes like AY36 and DB86 are also utilized in the textile sector^15,16^. In addition to being used by the textile industry, AY36 and DB86 dyes have additionally been reported to have been utilized in the production of pigment, shoe polish, laundry detergent, cleaning agents, and soap. However, due to their carcinogenic effects, their utilization in the food industry is strictly banned^15,16^. AY36 and DB86 dyes are colorants for acrylic fibres that are extensively utilized in the printing and textile industries. They may exist at different pH levels in cationic and zwitterionic forms^15,16^. These dyes have various industrial applications, but their runoffs pose a risk to plants, animals, aquatic life, and the ecosystem as a whole^2,16^. Cancer, allergies, and skin disorders are reportedly among the health problems that can arise from individuals drinking and absorbing dye-contaminated water^15,16^.

A critical characteristic of the ecological management and sustainable development as industries continues to develop globally and producing huge amount of wastewater containing various pollutants is the treatment of industrial effluent. In response to this challenge, the search for advanced and effective treatment technologies, which is joined with developing trends in process optimization and regulatory compliance is influencing the treatment of industrial effluents. With the growing intricacy and unpredictability of industrial runoffs, there is a rising demand for multipurpose and robust treatment solutions capable of addressing a huge variety of pollutants^17^.There have been reports of the effective removal of these dyes from single and binary aqueous solutions using techniques such as adsorption/ biosorption^6^, coagulation/flocculation^18^, the electro-Fenton process^19–21^, catalytic oxidation^22^, ultrafiltration^23^, ozonation^24–27^, biological activation^28^, and microbial fuel cells^29^, before these synthetic dyes such as AY36 and DB86 from industrial effluents are being released into the environment^15,16^.

According to several studies, one of the most often used ways to remove effluents such as metals and dyes from industrial wastes is the biosorption or adsorption of dyes onto activated carbon^4,7,30–37^. The ability of various agricultural wastes, such as tree fern, bark, rice husk, cotton waste, sugarcane dust, and watermelon wastes, as well as other low-cost adsorbents to adsorb dyes and remove various basic and acid dyes, has also been explored^4,7,38–46^. Numerous studies have also documented the removal of dyes from wastewater using biochar made from various biomasses, such as animal waste, plant and algal biomass, residential and forest waste, sewage sludge, etc^47–50^. Using biochar for wastewater treatment is favourable because of its large surface area and high number of surface functional groups (FGs)^47,51–56^. The use of biochar as an adsorbent has several benefits, including being economical, simple to use, and eco-friendly. Various precursors are readily accessible for making biochar, and it has also been reported that biochar has the potential to be recycled and has a better adsorption capacity than other common adsorbents^2,51,56,57^.

Therefore, the goal of this study is to investigate a novel approach to the removal of hazardous dyes (AY36 and DB86 dyes) from aqueous environments using *Delonix regia* biochar-sulphur (DRB-S), which was made from *Delonix regia* seed pods (DRSPs). *Delonix regia* is a leguminious plant of the sub-family *Caesalpinoideae*. It is generally grown as an orbnamental or agroforestry tree and produces a huge amount of seed pods and seed during the fruiting season and at present-day are not used. There are not any documented tonnage of seeds produced yearly, possibly due there has not been no reported viable value. The seeds of this plant rots and are wasted owing to that they are put into use^58^. DRB-S is an environmentally acceptable and reasonably priced adsorbent with many surface FGs. To the best of the authors’ knowledge, this is the first research to employ DRB-S to remove the dyes AY36 and DB86, which makes it unique. The batch biosorption studies for confiscating AY36 and DB86 dyes from industrial untreated dye effluents were characterized, and the optimization of the various parameters and factors was highlighted. Furthermore, an estimate of the thermodynamics, kinetics, and adsorption isotherms was considered. The basis for this recent study was to establish the use of DRB-S as one of the effective activated biomass-based biosorbents for the removal of AY36 and DB86 dyes utilizing the biosorption technique from industrial untreated dye effluents before being discharged into the nearby water bodies (that will sequentially contaminate the aquatic environment as well as the entire environment), and this will enormously be of great benefit in extenuating environmental contamination from industrial untreated dye effluents and contribute significantly to the desired safety and sustainability of our environment.

## Materials and methods

### Instrument and materials

DPSPs were collected from a local area in Alexandrian and utilised as the raw material to create DRB-S, an adsorbent substance. Sulfuric acid (H_2_SO_4_, Purity 98%), AY36 and DB86 dyes were obtained from Sigma Aldrich, USA. Concentrations were measured using an analytical Jena digital spectrophotometer (SPEKOL1300 UV/Visible spectrophotometer) in conjunction with 1 cm optical path glass cells, a shaker (JSOS-500) for mixing procedures, and a pH metre (JENCO 6173) for pH surveys. The adsorption-desorption isotherm of DRB-S was measured in the N_2_ environment. Using an instrument (BELSORP – Mini II, BEL Japan, Inc.), the surface area, pore size and pore distribution of DRB-S were determined^59,60^. Monolayer volume (*V*_*m*_) (cm^3^ (STP), surface area (S_BET_) (m^2^/g), average pore diameter (MPD) (nm), total pore volume (*p*_*0*_/*p*_*0*_) (cm^3^/g) and energy constant (*C*) values of DRB-S were obtained by modeling of the adsorption-desorption graph. The microporous surface area (*S*_*mi*_), mesoporous surface area (*S*_*mes*_), mesoporous volume (*V*_*mes*_), and microporous volume (*V*_*mi*_) of DRB-S were calculated by the Barrett–Joyner–Halenda (BJH) model. The calculations were carried out using the BELSORP analysis software. Using the BJH approach, the pore size dispersion was also ascertained from the desorption isotherm^61^. An investigation of the form of the biochar surface was conducted using a scanning electron microscope (SEM; QUALITY 250). Fourier Transform Infrared (FTIR) spectroscopy (VERTEX70) and the ATR unit model V-100 were used to investigate the FGs on the surface of DRB-S. IR-observable FGs on the DRB-S surface were identified in the 400–4000 cm^–1^ wavenumber region using FTIR spectroscopy in combination with the platinum ATR unit. Employing the SDT650-Simultaneous Thermal Analyzer apparatus, thermal analyses were conducted at a ramping temperature of 10 °C/min throughout a temperature range of 50–1000 °C.

### DRB-S preparation

DPSPs were extensively cleansed with tap water many times to remove any dust, and they were thereafter dried in a furnace at 115 °C for twenty-four hours before being ground and pulverised. A total of 120 g of powdered DPSPs was heated at 260 °C in 600 mL of 85% H_2_SO_4_ solution for 6 h, then diluted with distilled water, filtered and then washed with distilled water until pH 7. The DRB-S was then cleaned with EtOH and dried at 115 °C in a furnace. Biochar with the designation DRB-S was produced as a consequence of this reaction.

### Batch adsorption experiment

**Fig. 1:**
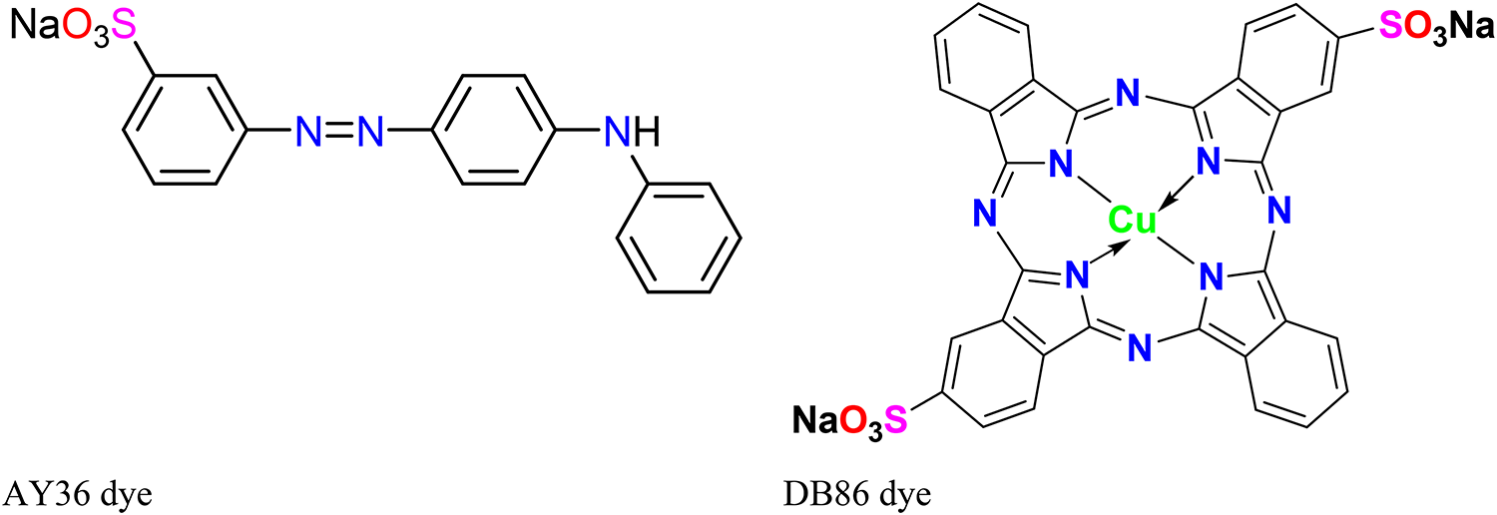
Structure of AY36 and DB86 dyes.

A batch adsorption experiment was used to assess the sorption capacity, thermodynamic, and kinetic properties of DRB-S. A series of 300 mL Erlenmeyer flasks were filled with 100 mL of AY36 (Metanil Yellow, C_18_H_14_N_3_NaO_3_S) and DB86 (Solvent Blue 38, C_32_H_14_O_6_N_8_S_2_CuNa_2_) dyes (Fig. 1) solutions at various starting concentrations and DRB-S at various weights were shaken for a predetermined amount of time at 200 rpm. Solution pHs were raised or lowered to the appropriate levels with 0.1 M NaOH or HCl. Furthermore, during the adsorption equilibrium investigations, the pH of the solution was maintained at the intended level. Taking a sample (0.1 mL) from the solution at regular intervals (removed from the adsorbent) allowed for the determination of the AY36 and DB86 dye concentration using a spectrophotometer set at λ_max_ = 594 and 615 nm, respectively. All experiments were repeated three times with a difference of less than 2.5%, and only the average values were used in the calculations. The *q*_t_ of DRB-S was calculated using Eq. (1).

1 $${q_t}=\frac{{\left( {{C_0}~ - ~{C_t}} \right)}}{W}V$$

where *C*_*0*_ (mg/L) is the AY36 and DB86 dyes initial concentration; *C*_*t*_ (mg/L) is the remaining AY36 and DB86 dyes concentration at the end of time *t*; *q*_*t*_ (mg/g) is the adsorption capacity of DRB-S at time *t*; *W* (g) is the mass of the DRB-S and *V* (L) is the volume of the AY36 and DB86 dyes solutions.

To examine the impact of pH on the adsorption of AY36 dye and DB86 dye ions by DRB-S, studies were achieved at different pH values (1.11 to 13.15) and (1.56 to 13.32), respectively, by adding 0.1 g DRB-S to 100 mL of solutions containing 100 ppm of AY36 dye and 50 ppm of DB86. The mixtures were agitated for 150 min at 200 rpm when the mixtures were at room temperature.

AY36 dye and DB86 dye solutions with varying initial concentrations (50–150 ppm) were made, and isotherm measurements and the effect of DRB-S dose on the adsorption of AY36 dye and DB86 dye ions were investigated. Intervals between 0.75 and 1.75 g/L of DRB-S doses and AY36 dye and DB86 dye solutions with diverse starting concentrations were used to measure the AY36 dye and DB86 dye concentrations. The mixtures were agitated at 200 rpm and 25 °C. Every adsorption investigation was carried out in triplicate, and the results are presented as an average.

## Results and discussion

### DRB-S characterization

**Fig. 2:**
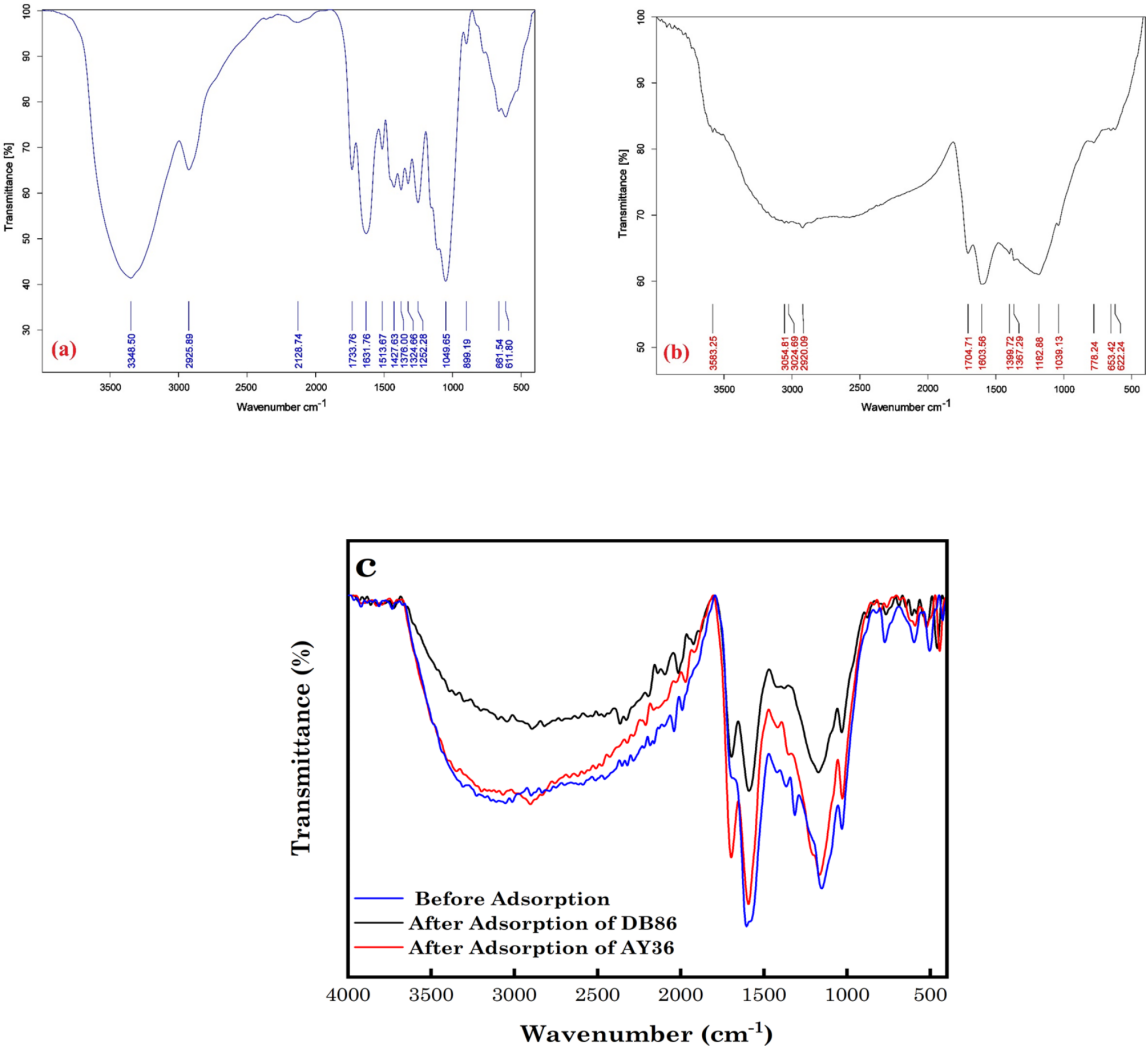
FTIR graphs of (a) DPSPs, (b) DRB-S and (c) before and after adsorption of AY36 and DB86 dyes to DRB-S.

Using FT-IR spectroscopy, the FGs present on the surface of the resulting DRB-S adsorbent were identified. The FTIR graph of the raw DPSPs and the FTIR graph of the DRB-S were compared, as shown in Fig. 2a, b. The FT-IR spectra of the materials show changes in their FGs. The stretching oscillation of the O-H present in the DPSPs and DRB-S is demonstrated by the band between 3583.25 and 3348.50 cm^–1^ (Fig. 2). The presence of –CH_2_ stretching groups in DPSPs is suggested by the high absorption peaks between 2925.89 cm^–1^ (Fig. 2a). These groups were enlarged in DRB–S and appeared at 2920.09 cm^–1^ (Fig. 2b). The C=O stretching of the ester groups in the DPSPs is responsible for the high absorption band at 1733.76 cm^–1^ (Fig. 2a). This band was later transformed into a carboxyl group in DRB-S at 1704.71 cm^–1^ (Fig. 2b). Nevertheless, the strength at 1704.71 cm^–1^ increased when DRB-S was compared to raw DPSPs, indicating that sulphuric acid treatment may increase the carbonyl (C=O) group. The bands at 1631.76 cm^–1^ suggest that the *β*-ketone’s C=O stretching oscillation was nearly existent in the DPSPs. This oscillation shifted to 1603.56 cm^–1^ in DRB-S with high intensity, and it might also be a stretching vibration of –C =C– in DRB-S (Fig. 2b). The DPSPs’ C-O FG is shown by the peaks at 1513.67–1252.28 cm^–1^. This group was replaced by the band at 1399.72 and 1367.29 cm^–1^ in DRB–S, which displayed the sulfonyl group (S =O) stretching vibration (Fig. 2b). Additionally, the development of peaks at 1182.88 and 1039.13 cm^–1^ was facilitated by the dehydration process with H_2_SO_4_. These peaks resulted from the production of –SO_3_H and S =O groups in DRB-S. These bands show that the DPSP treatment with H_2_SO_4_ results in the creation of the DRB-S. The DPSPs showed a more noticeable rise in the –C–O–C– asymmetric stretching FG at 1049.65 cm^–1^ (Fig. 2a), compared to DRB-S, which showed a partly weaker increase^54–57^. As observed in Fig. 2c, there was a red shift in most of the peaks of –C=C–, –SO_3_H, O–H and S=O after the adsorption of AY36 and DB86 to DRB-S with associated intensity changes. This indicates that the uptake of the dye molecules was attributed to these FGs.

**Fig. 3:**
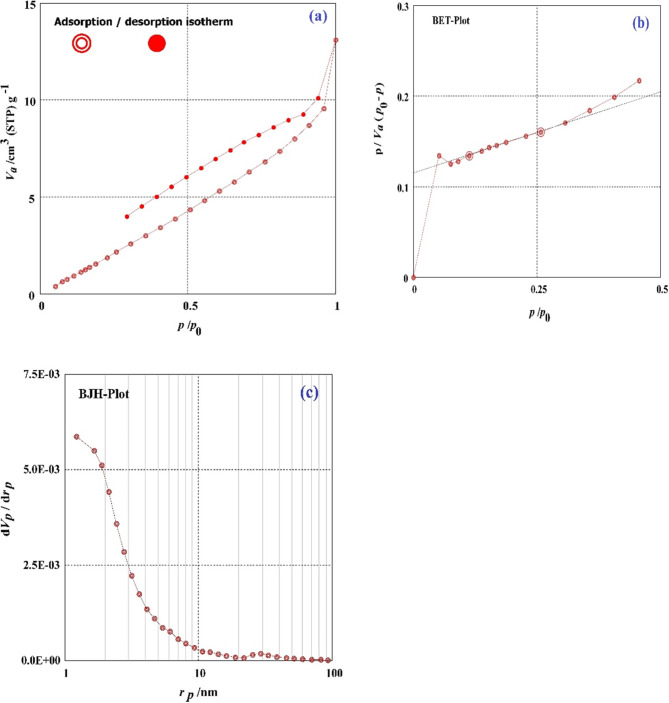
(a) Graph of N_2_ Adsorption-Desorption, (b) Graph of the BET, (c) Graph of the BJH of the DRB-S.

To find out how H_2_SO_4_ affected the DRB-S’s surface characteristics, the N_2_ adsorption-desorption isotherm of the DRB-S was studied. The BET and BJH methods were used to compute the specific surface and mesopore areas, respectively. Figure 3 shows the textural properties of DRB-S, including BET-specific surface area, mass of mesopores, mesopore area, total volume of pores, mesopore distribution peak, average pore diameter, and monolayer volume. The DRB-S has a relatively tiny BET-specific surface area of 14.745 m^2^/g. DRB-S had a monolayer volume value of 3.3878 cm^3^ (STP) g^–1^. DRB-S has a total volume value of 1.8975 × 10^–2^ cm^3^/g. DRB-S had mean pore diameters of 5.1474 nm. The values of 14.923 m^2^/g, 2.1973 × 10^–2^ cm^3^/g, and 1.22 nm were found to be the mesopore volume, meso surface area, and mesopore distribution peak values of DRB-S, respectively.

**Fig. 4:**
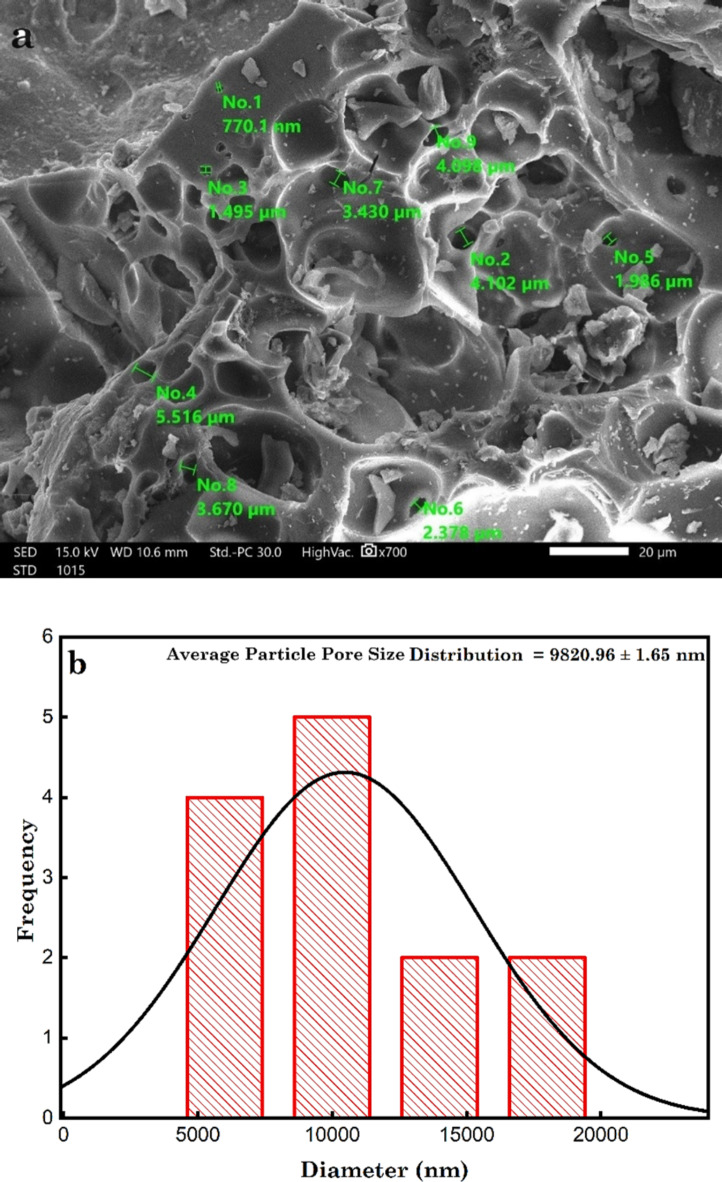
(a) SEM image of DRB-S using High vacuum SEM at magnification ×700 and 15.0 kV, and (b) particle pore size distribution of DRB-S.

The DRB-S is shown in SEM pictures in Fig. 4a, where it is clear that it is clean and impurities-free. The DPSPs’ pore structure remained unharmed by the intense sulfuric acid treatment. The particle pore size distribution shows that the particle pore sizes were within the range of 4072–17,200 nm, and the determined average particle pore size distribution of the DPKB-S was 9821 ± 1.65 nm according to ImageJ assessment (Fig. 4b).

The DRB-S adsorbent chemical composition was studied using scattered X-ray spectrometry (EDX). The percent of each element is presented in Table 1, which indicates that, in addition to carbon mass %, which makes up 56.26% of the sample, there are around 41.50 and 0.37% of oxygen and sulphur, respectively.

**Table 1:** EDX results of prepared DRB-S.

Elements	DRB-S	
	Mass%	Atom%
C	56.26 ± 0.33	63.61 ± 0.37
O	41.50 ± 0.67	35.22 ± 0.57
S	0.37 ± 0.04	0.16 ± 0.02
Na	1.53 ± 0.10	0.90 ± 0.06
Ca	0.34 ± 0.04	0.12 ± 0.01
Total	100.00	100.00

**Fig. 5:**
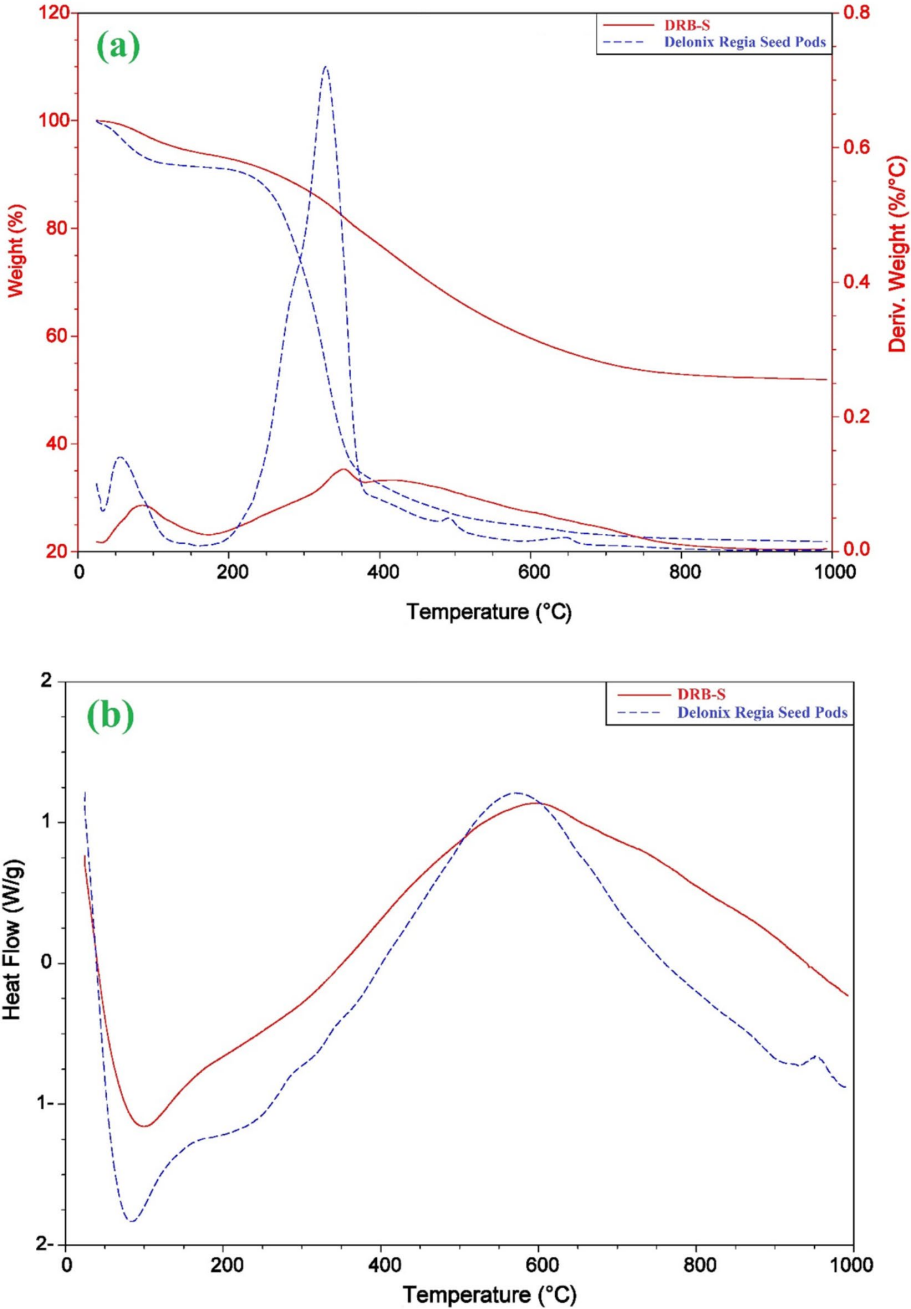
Graphs of (a) DTA and TGA, and (b) DSC of the DPSPs and DRB-S.

The impact of structural variations on the operating temperature and degradation behaviour of the DRB-S samples and raw date palm kernel was assessed using thermal gravimetric analysis (TGA). Every sample was heated from 50 to 1000 °C in a N_2_ atmosphere. Figure 5 displays the TGA, Differential Thermal Analysis (DTA) and Differential Scanning Calorimetry (DSC) analytical curves for DPSPs and DRB-S. The first weight reduction was caused by the evaporation of water in the raw DPSPs and DRB-S, and it peaked before 150 °C. Raw DPSPs and DRB-S lost weight as a result of the breakdown of many acidic oxygen FGs that occurred as the temperature rose beyond 150 °C. Moreover, acidic groups break down at different temperatures. For example, phenol breaks down at a greater temperature than lactones, anhydrides, and carboxylic groups. Raw DPSPs exhibit a high weight loss at temperatures up to 328.5 °C and the final weight loss occurs between 328 and 450 °C. DRB-S shows three weight losses at temperatures between 25 and 150, 150–380 and 380–950 °C, which explains the higher stability of DRB-S compared to the raw DPSPs. TGA curve of DRB-S converged at temperatures > 400 °C due to carbon breakdown in biomass. At the finishing temperature, various weight loss percentages of 78.03 and 48.06% were obtained for raw DPSPs and DRB-S, respectively, indicating the greater stability of DRB-S.

The DTA graph of DRB-S and raw DPSPs is illustrated in Fig. 5a. The DTA curve of the raw DPSPs (blue) peaked at two points at temperature (*T*_*f*_, 58.10 and 328.30 °C), while the curve of DRB-S (blue) peaked at three points at temperature (*T*_*f*_, 85.40, 350.06 and 454.66 °C) (Fig. 5a). As can be seen from the DTA curve to produce DRB-S adsorbents from raw DPSPs, dehydration of raw DPSPs (blue) showed two well-resolved degradation bands. The degradation bands of raw DPSPs (blue) decreased from three to two at higher temperatures after treatment with 85% H_2_SO_4_, demonstrating that the degree of degradation was strongly affected by H_2_SO_4_ treatment.

**Fig. 6:**
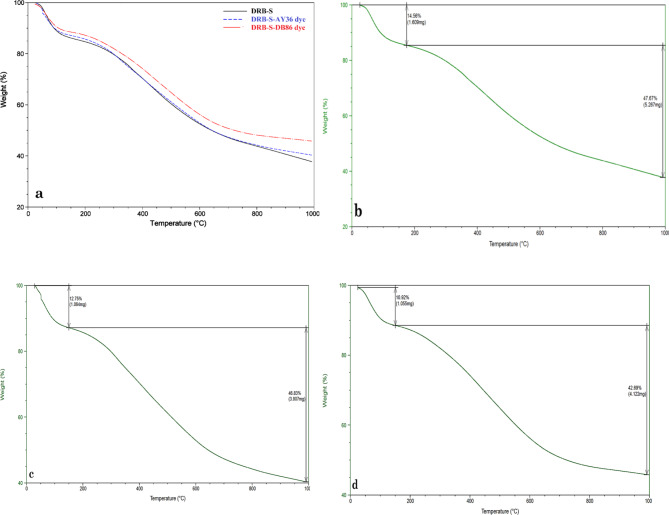
TGA of (a) DRB-S, before and after of AY36 and DB86 dyes (b) DRB-S, (c) DRB-S-AY36 and (d) DRB-S-DB86 dyes after adsorption.

DSC may be used to compare materials based on thermal transitions. Figure 5b depicts the DSC graph of DRB-S (red) and raw DPSPs (blue). The crystallisation temperatures (*T*_C_) of DPSPs are 72.76 °C, while DRB-S displays *T*_C_ values of 82.11 °C. When the temperature rises, DRB-S melts at 587.95 °C, while DPSPs melts at 565.13 °C. A lower *T*_m_ was shown by DPSPs, whereas the highest *T*_m_ was shown by DRB-S. The grains became more crystalline due to the higher transitional temperatures, improving their structural stability and resistance to gelatin disintegration. Based on Fig. 6b, a weight loss was observed in the TGA analysis of before and after adsorption of AY36 and DB86 dye molecules to DRB-S at 150, 328, 450, and 950 °C. At 150 and 950 °C, it was observed that the DRB-S sorbent had a greater weight loss of 14.56% and 47.67% (Fig. 6b). After the adsorption of AY36 and DB86 dyes to DRB-S, there was a decrease in weight loss of 12.75% and 10.92% noticed at 150 °C. At 950 °C, there was also a reduced weight loss of 46.83% and 42.69% observed after the adsorption of AY36 and DB86 dyes to DRB-S (Fig. 6c, d). These reduced weight loss after adsorption of AY36 and DB86 dyes at 150 and 950 °C were ascribed to the decomposition of the dye molecules.

**Fig. 7:**
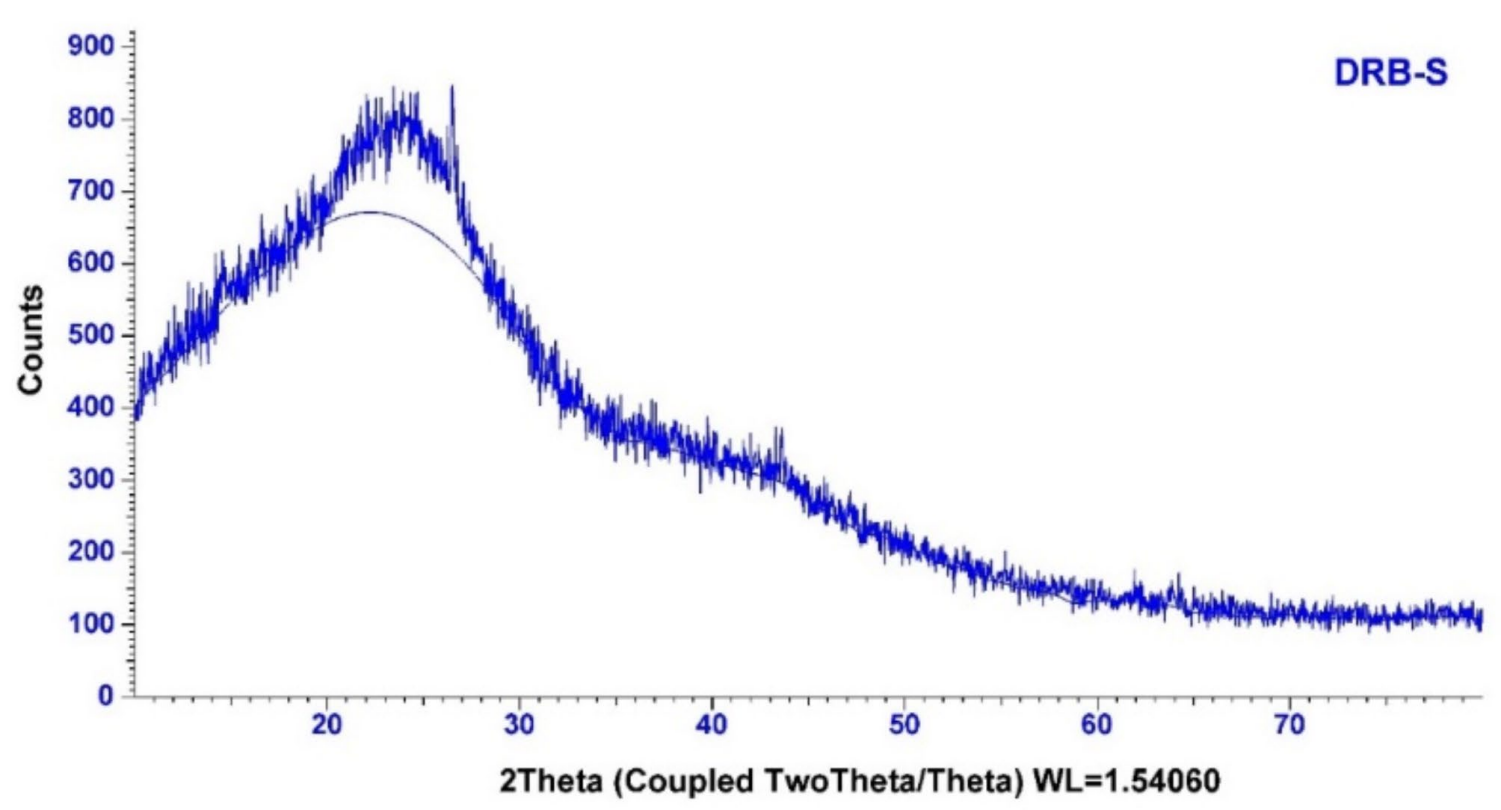
XRD graph of fabricated DRB-S biochar.

The DRB-S XRD is shown in Fig. 7 and shows an amorphous carbon structure with arbitrarily oriented aromatic sheets. A tiny and broad peaks located at 2Ɵ = 44.6$\:^\circ\:$ and 21.7$\:^\circ\:$ which were indexed at 101 and 002 planes of amorphous carbon or common feature of a non-crystallite structure of activated carbon as well as bear a resemblance to the graphitic hexagonal structure of carbon based materials^66–69^. The average crystallite size (D_hkl_) of the miniscule powdered particles in the hkl direction was obtained using the Debye Scherrer equation (Eq. 2).

2 $${D_{hkl}}=\frac{{K\lambda }}{{{\beta _{hkl}}\cos \left( {{\theta _{hkl}}} \right)}}$$

Where *h*,* k*,* l*,* K*, $\lambda$, $\theta$ and $\beta$ represent the miller indices, the shape factor (0.9), the wavelength of the diffraction beam (0.15406 nm), Bragg angle and the full width half medium (FWHM) of the X-ray diffraction peaks (radians)^70,71^. The crystalline size of the 002 and 101 peaks were dteremined to be 0.34 and 0.50 nm.

Equations 3–7 show the average crystallite lattice parameters of carbon material (interplanar spacing of the aromatic layers of D_002_ and D_100_), crystallite height of the plane 002 (L_c_), crystallite diameter of the plane 101 (L_a_) and the average number of effective aromatic layer per carbon crystallite (N_ave_) were obtained employing the Bragg’s equation and the empirical equations obtained from Scherrer equation. 

3 $${D_{002}}=~\frac{\lambda }{{2\sin {\theta _{002}}}}$$

4 $${D_{101}}=~\frac{\lambda }{{2\sin {\theta _{101}}}}$$

5 $${L_c}=~\frac{{{K_c}\lambda }}{{{\beta _{002}}\cos {\theta _{002}}}}$$

6 $${L_a}=~\frac{{{K_a}\lambda }}{{{\beta _{101}}\cos {\theta _{101}}}}$$

7 $${N_{ave}}=\frac{{{L_c}}}{{{D_{002}}}}+1$$

${\theta _{002}}$, ${\theta _{101}}$, ${\beta _{002}}$, ${\beta _{101}}$, *K*_*c*_*and K*_*a*_ are the X-ray diffraction peaks at 002 and 101, FWHM of the 002 and 101 peaks and K shaped factor constants for 002 (0.89) and 101 (1.84) peaks^72,73^. In Table, the determined D_002_ and D_101_ values were 0.410 (samples have a lower-level ordered crystallite unit relative to hite) and 0.203 nm. While the determined L_c_ and L_a_ values were 0.338 and 1.022 nm. The extensive diffraction at 002 peak noticed for DRB-S suggested that the material was extremely disordered, moderately crystallized and tically graphite in phase morphology. Hence this peak is assigned to the graphitic planes. The interplanar spacing f the 002 peak was large to the value of 0.335 nm projected for ideal graphite (JCPDS 00-056-0159). Hence the D_002_ value of 0.410 nm was anticipated to be an effective electrode materials for storage applications. It was also observed that the determined L_a_ value was larger than the obtained L_c_ value from peaks 101 and 002. This was suggestive of the growth of the graphitic structure being on the planes^74–76^.

**Table 2:** Determined interlayer spacing anf Microcrystallite dimesions of DRB-S.

D_002_ (nm)	D_101_ (nm)	L_c_ (nm)	L_a_ (nm)	*N*_ave_
0.410	0.203	0.338	1.022	1.989

###  pH effect

**Fig. 8:**
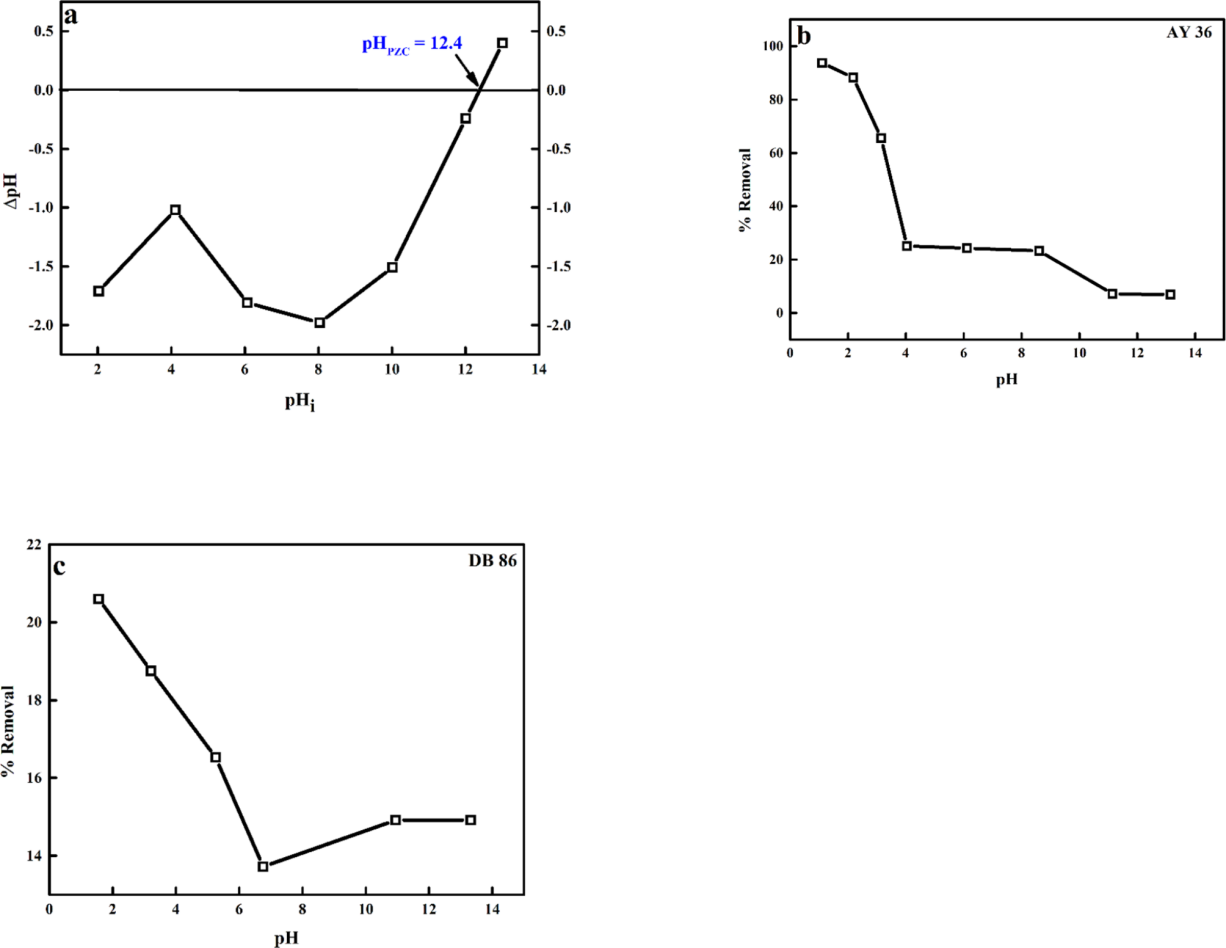
(a) pH_PZC_ of the prepared biochar-S (DRB-S), (b) pH impact on the biosorption of AY36 dye and (c) DB86 dye onto DRB-S at room temperature.

A critical parameter that controls the sorption of dye to biosorbents is the pH effect (Fig. 8). Dyes are known to occur in an ionic form in a water-soluble solution and the degree of sorption on the surface of the biosorbent is impacted by the biosorbent surface charge, which in turn impacts the pH of the aqueous solution^77,78^. Also, the electrostatic interaction between the biosorbent surface and the dye molecules is enhanced by the variation of the point of zero charge (pH_PZC_) of the biosorbent through the surface variation. The biosorbent surface sites show a positive and negative charge when pH < pH_pzc_ and pH > pH_pzc_^79^. The pH_PZC_ of the prepared biochar was determined to be 12.4 (Fig. 1a). Figure 1b and c show the percentage (%) removal of AY36 (94%) and DB86 (21%) dyes confiscated to the biochar as the pH was varied (pH 1.1–13.2). As observed in Fig. 1b and c, the % removal of AY36 and DB86 dyes sorbed to the biochar decreased with increasing pH, with the optimum % removal of both dyes noticed at pH 1.1. For AY36, this phenomenon was ascribed to the electrostatic attraction between the increased positively charged sites on the biochar surface (hydrogen ions - H^+^), and the cationic dye molecules. With further increase in the solution pH (basic condition), the surface charged sites on the biochar became negatively charged (hydroxyl ion-OH^−^) and this led to the electrostatic repulsion between the cationic dye molecules and the negatively charged surface sites on the biochar (excess OH^–^). A similar trend was observed in the studies of El-Nemr et al.^80^, Garg et al.^81^ and Thirunavukkarasu et al.^82^.

### Dosage effect

**Fig. 9:**
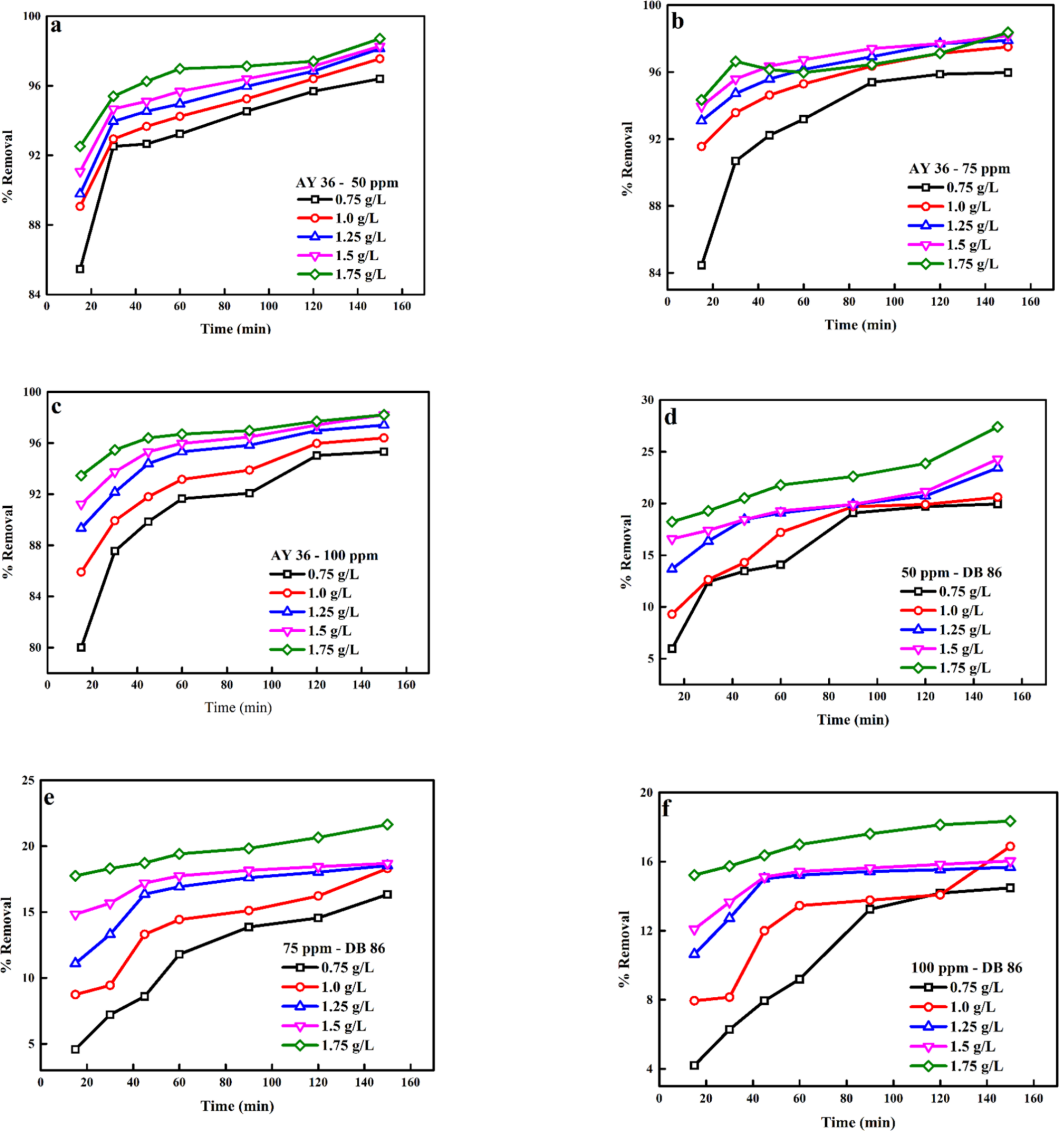
Effect of biosorbent dosage on the confiscation of (a) AY36 dye—50 ppm, (b) AY36 dye—75 ppm, (c) AY36 dye—100 ppm, (d) DB86 dye—50 ppm, (e) DB86 dye—75 ppm and (f) DB86 dye—100 ppm.

A critical factor that should be considered and which influences the extent of biosorption of dye is the biosorbent dosage^83^. The % of AY36 dye and DB86 dye confiscated to the prepared biochar (Fig. 9a–f) was observed to increase significantly as the biosorbent dosage and time were improved from 0.75 to 1.75 g/L and 10–150 min for 50–100 ppm of both dyes (Tables S1–S4). This occurrence was ascribed to the intensification of the number of available active sites on the biochar surface as the biochar dosage was increased, thereby increasing the % of dye molecules confiscated to the prepared DRB-S^84,85^. As shown in Tables S1–S4, the adsorption capacity was raised by raising the initial dye concentrations and lowered by increasing the dose of DRB-S.

### Initial concentration effect

**Fig. 10:**
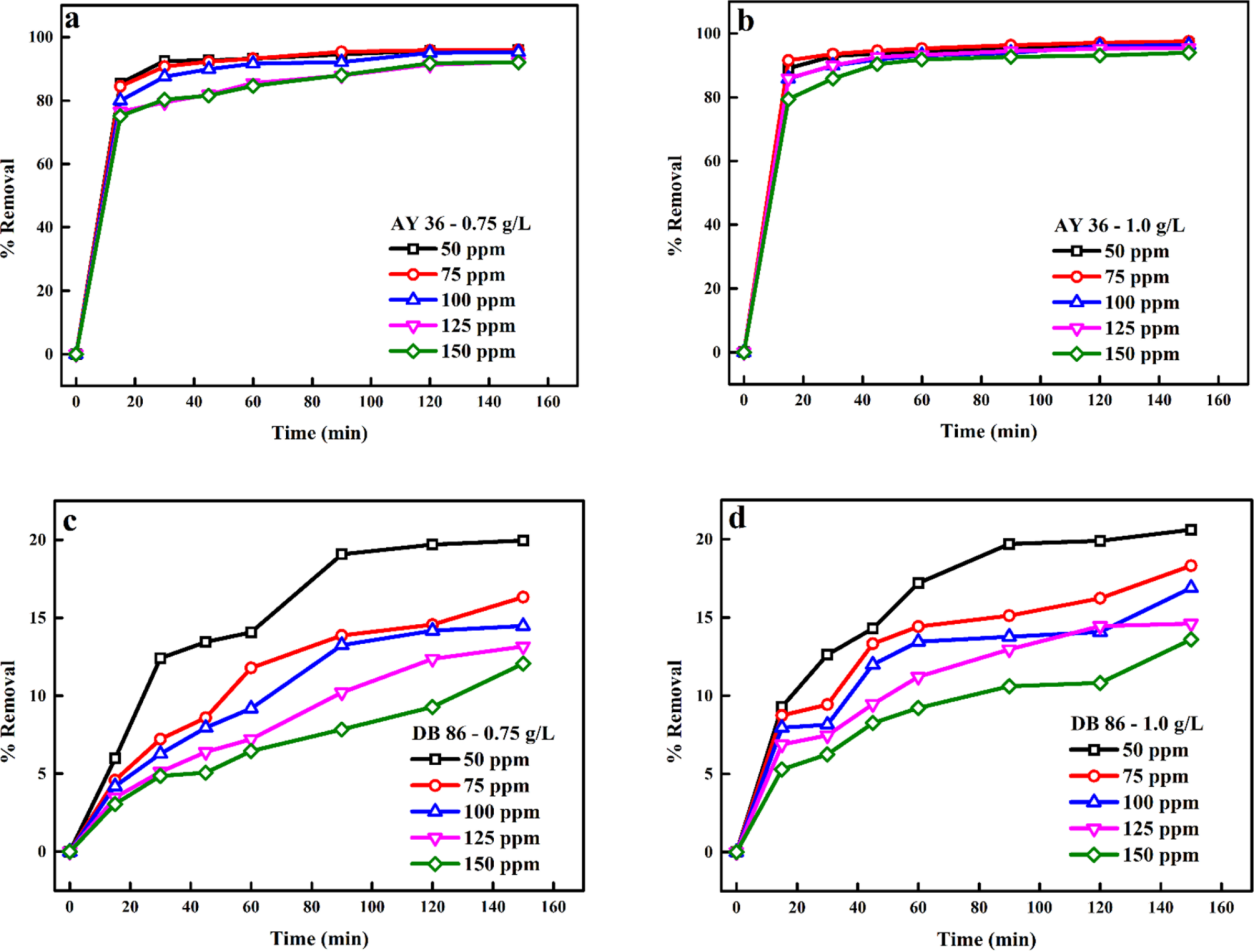
Influence of initial concentrations on the removal of (a) AY36 dye using 0.75 g/L biochar dosage, (b) AY36 dye using 1.0 g/L biochar dosage, (c) DB86 dye using 0.75 g/L biochar dosage and (d) DB86 dye using 1.0 g/L biochar dosage.

The initial sorbate concentration describes the sorbate molecules partitioning behaviour among the solid biosorbent and the mass liquid solution at equilibrium^86^. The impact of the starting dye concentrations (50–150 ppm) on the biochar’s confiscation efficiency is depicted in Fig. 10a–d. As reaction interaction time was increased from 0 to 150 min, it was shown that the optimal removal of AY36 and DB86 dyes decreased with an increase in the starting dye concentrations (Tables S1, S2). It was also noticed that the % of AY36 and DB86 dyes removed was gradually reduced and equilibrium was attained at 30 and 90 min of reaction interaction, owing to the saturation of available accessible active sites on the biochar surface at elevated dye concentrations. At low concentrations, the ratio of the available, accessible active sites on the surface of the biochar was high, hence leading to the increased diffusion of dye molecules from the solution to the active sites of the biochar. This boosted the driving force of the concentration gradient^87–89^.

### Kinetic models

Kinetic research defines the reaction rates that assess the contact time needed to accomplish the biosorption equilibrium. It is a crucial variable in every biosorption investigation. The rate and mechanism by which the contaminant is removed must be understood very precisely, and for this, the kinetic models explored in this study were the pseudo-first-order model (PFOM), pseudo-second-order-model (PSOM) and the intra-particle diffusion model (IDM) and film diffusion model (FIM)^90^. The linearized form of these different models is given by Eqs. (8)–(11).

8 $$\log {q_e} - {q_t}=\log {q_e} - {K_1}t$$

9 $$\frac{t}{{{q_t}}}=\frac{1}{{{K_2}q_{e}^{2}}}+\frac{t}{{{q_e}}}$$

10 $${q_t}={K_{IDM}}{t^{0.5}}+C$$

11 $$\ln \left( {1 - F} \right)~={\text{~}} - {K_{FD}} \times t$$

**Fig. 11:**
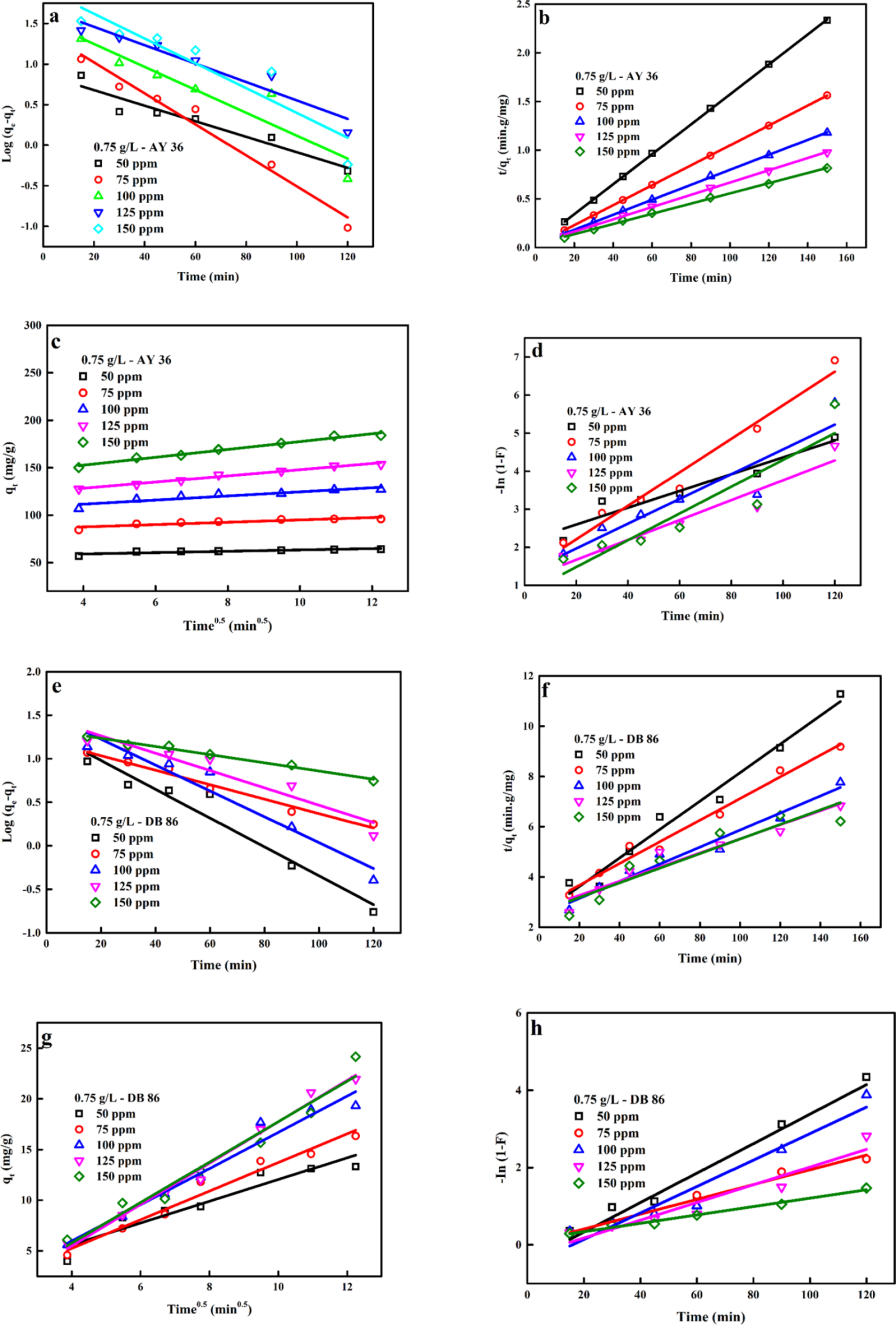
Linear plots of (a–d) PFOM, PSOM, IDM and FIM for the biosorption of AB36 dye, (e–h) PFOM, PSOM, IDM and FIM for the biosorption of DB86 dye.

*K*_*1*_, *K*_*2*_ and K_IDM_ are the PFO rate constant (min^− 1^), PSO rate constant (g.mg^− 1^.min^− 1^), ID rate constant (mg.g^− 1^.min^1/2^), and the *C* is the intersection of the line with the ordinate axis. The value of *C* gives an idea about the boundary layer thickness. Meanwhile, F and KFD signify fractional attainment of equilibrium and film diffusion rate coefficient (L/min). A plot of ln (1-F) against t, with a zero intercept, indicates that thadsorption procedure is controlled by the diffusion of the liquid film around the adsorbent^91–93^. The plots of the linearized form of all models are given in Fig. 11. According to the result provided in Tables 3 and 4, the PSOM best defines the biosorption of AY36 and DB86 dyes to the nanomaterial based on the determined correlation coefficients (*R*^2^) of this model ($>0.99 - {\text{close~to~unity}}$), which was higher than the PFOM. Also, the experimental *q*_e_ values of this model were close to the calculated *q*_e_ values. From the calculated parameters of IDM and FIM in Tables 5 and 6, the IDM and FIM plots did not offer a straight line that goes through the origin of the plots, and the *R*^2^ values were less than those of the PSOM. This indicated that the diffusion of dye molecules in the liquid film around the prepared biosorbent was not rate-defining steps and diffusion but might advance the adsorption process at the jolt of the biosorption process. Hence, the biosorption process of both dyes to the biosorbent assumed a chemisorption process which involved the valency force through the exchange of electrons between the dye molecules and the prepared biosorbent^94^.

**Table 3:** Comparison of the PFO and PSO rate constants and calculated parameters for different initial AY36 dye concentrations.

Parameter			PFOM			PSOM			
DRB-S Dosage	AY36 (ppm)	*q*_*e*_ (exp.)	*q*_*e*_ (calc.)	*k*_*2*_ × 10^3^	*R* ^*2*^	*q*_*e*_ (calc.)	*k*_*2*_ × 10^3^	*h*	*R* ^*2*^
0.75 g L^–1^	50	64.27	7.45	22.11	0.923	64.94	6.83	28818.44	1.000
	75	95.97	25.42	43.99	0.974	98.04	4.15	39840.64	1.000
	100	127.10	34.05	32.68	0.878	129.87	2.09	35211.27	1.000
	125	153.62	48.67	26.25	0.925	158.73	1.06	26737.97	0.999
	150	184.07	84.55	35.24	0.854	192.31	0.84	30959.75	0.999
1.0 g L^–1^	50	48.78	4.56	16.81	0.958	49.26	9.56	23201.86	1.000
	75	73.13	6.71	24.87	0.984	73.53	8.64	46728.97	1.000
	100	96.40	16.37	2.72	0.921	98.04	3.69	35460.99	1.000
	125	119.31	19.28	33.62	0.985	120.48	3.94	57142.86	1.000
	150	141.00	23.10	26.02	0.917	142.86	2.33	47619.05	1.000
1.25 g L^–1^	50	39.25	3.27	15.66	0.936	39.53	12.93	20202.02	1.000
	75	58.73	4.96	28.56	0.949	59.17	13.41	46948.36	1.000
	100	77.93	8.93	25.79	0.962	78.74	6.58	40816.33	1.000
	125	97.06	10.62	23.26	0.940	98.04	4.95	47619.05	1.000
	150	114.13	26.43	42.84	0.988	116.28	3.54	47846.89	1.000
1.5 g L^–1^	50	32.76	2.32	15.43	0.933	32.89	18.41	19920.32	1.000
	75	49.09	2.49	20.27	0.987	49.26	20.40	49504.95	1.000
	100	65.47	2.67	19.11	0.965	65.79	8.99	38910.51	1.000
	125	81.56	5.55	21.65	0.940	81.97	9.42	63291.14	1.000
	150	97.07	8.89	20.50	0.889	98.04	5.45	52356.02	1.000
1.75 g L^–1^	50	28.20	1.51	13.36	0.819	28.33	27.33	21929.82	1.000
	75	42.16	1.43	7.60	0.580	42.19	23.02	40983.61	1.000
	100	56.11	2.96	18.88	0.942	56.50	16.32	52083.33	1.000
	125	70.11	4.61	23.03	0.949	70.42	11.93	59171.60	1.000
	150	83.95	6.80	20.73	0.845	84.75	7.25	52083.33	1.000

**Table 4:** Comparison of the PFO and PSO rate constants and calculated parameters for different initial DB86 dye concentrations.

Parameter			PFOM			PSOM			
DRB-S Dosage	DB86 (ppm)	*q*_*e*_ (exp.)	*q*_*e*_ (calc.)	*k*_*1*_	*R* ^*2*^	*q*_*e*_ (calc.)	*k*_*2*_ × 10^3^	*h*	*R* ^*2*^
0.75 g L^–1^	50	12.99	11.70	18.19	0.994	17.06	1.14	330.71	0.996
	75	16.65	18.43	22.57	0.990	24.04	0.63	363.54	0.997
	100	20.71	22.43	20.04	0.943	30.77	0.43	406.92	0.992
	125	22.56	26.22	19.81	0.955	35.84	0.30	386.92	0.973
	150	23.07	21.19	12.44	0.992	33.44	0.34	382.66	0.927
1.0 g L^–1^	50	10.26	7.60	24.64	0.998	11.81	3.69	514.32	1.000
	75	13.52	10.30	23.95	0.990	15.48	2.78	666.93	0.999
	100	15.85	9.67	18.19	0.989	17.67	2.64	823.93	0.996
	125	17.24	14.64	27.87	0.979	19.76	2.28	890.08	0.999
	150	18.52	17.68	24.41	0.960	22.47	1.31	663.44	0.995
1.25 g L^–1^	50	8.46	3.72	26.02	0.973	8.98	11.68	940.82	1.000
	75	10.99	5.50	29.48	0.984	11.76	7.48	1034.98	1.000
	100	12.85	4.25	13.82	0.868	13.33	11.18	1986.89	0.999
	125	14.70	3.32	20.27	0.790	15.17	7.55	1738.53	0.997
	150	15.15	4.64	23.26	0.952	15.75	9.39	2329.37	1.000
1.5 g L^–1^	50	7.19	1.64	11.28	0.927	7.30	19.74	1053.52	0.997
	75	9.36	2.46	24.64	0.989	9.65	19.97	1860.81	1.000
	100	10.84	2.65	20.73	0.894	11.17	15.72	1963.09	1.000
	125	12.46	3.59	15.66	0.919	12.87	9.63	1595.91	0.999
	150	13.11	4.34	16.58	0.964	13.64	7.90	1470.37	0.998
1.75 g L^–1^	50	7.11	2.38	13.36	0.964	7.38	12.58	684.98	0.994
	75	9.16	2.15	15.20	0.964	9.38	16.57	1457.94	0.998
	100	10.60	2.67	19.11	0.982	10.91	15.23	1810.94	0.999
	125	11.69	2.26	20.04	0.975	11.93	19.52	2779.32	1.000
	150	13.02	2.90	25.79	0.984	13.33	18.32	3256.27	1.000

**Table 5:** Comparison of the IDM and FIM rate constants and calculated parameters for different initial AY36 dye concentrations.

DRB-S dosage	AY36 (ppm)	IDM			FIM	
		*K*_*dif*_	*C*	*R* ^*2*^	*K*_*FD*_	*R* ^*2*^
0.75 g L^–1^	50	0.708	56.240	0.778	0.022	0.923
	75	1.238	82.553	0.826	0.044	0.974
	100	2.163	102.820	0.854	0.033	0.878
	125	3.275	115.160	0.985	0.026	0.925
	150	4.131	136.200	0.978	0.035	0.854
1.0 g L^–1^	50	0.438	43.544	0.913	0.017	0.958
	75	0.514	67.210	0.956	0.025	0.984
	100	1.173	82.968	0.926	0.027	0.925
	125	1.313	104.910	0.851	0.034	0.985
	150	2.311	115.770	0.783	0.026	0.917
1.25 g L^–1^	50	0.333	35.265	0.880	0.016	0.936
	75	0.334	54.908	0.954	0.029	0.949
	100	0.722	69.768	0.896	0.026	0.962
	125	1.015	85.682	0.824	0.023	0.940
	150	1.552	97.532	0.807	0.043	0.988
1.5 g L^–1^	50	0.239	29.899	0.878	0.015	0.933
	75	0.230	46.420	0.923	0.020	0.987
	100	0.503	59.592	0.917	0.019	0.965
	125	0.557	75.290	0.826	0.022	0.640
	150	0.990	86.000	0.748	0.020	0.889
1.75 g L^–1^	50	0.172	26.141	0.838	0.013	0.819
	75	0.150	40.123	0.736	0.008	0.580
	100	0.283	52.808	0.890	0.019	0.942
	125	0.460	64.998	0.784	0.023	0.949
	150	0.696	76.047	0.824	0.021	0.845

**Table 6:** Comparison of the IDM and FIM rate constants and calculated parameters for different initial DB86 dye concentrations.

Sorbent dose	DB86 (ppm)	IDM			FIM	
		*K*_*dif*_	*C*	*R* ^*2*^	*K*_*FD*_	*R* ^*2*^
0.75 g L^–1^	50	1.039	0.567	0.983	0.018	0.994
	75	1.464	− 0.503	0.981	0.023	0.990
	100	1.840	− 1.309	0.994	0.020	0.984
	125	2.070	− 2.528	0.997	0.020	0.955
	150	1.952	− 2.096	0.986	0.013	0.992
1.0 g L^–1^	50	0.637	3.044	0.919	0.029	0.998
	75	0.808	4.183	0.964	0.024	0.990
	100	0.879	5.393	0.963	0.018	0.989
	125	1.023	5.559	0.957	0.028	0.979
	150	1.288	3.425	0.987	0.024	0.960
1.25 g L^–1^	50	0.323	4.865	0.851	0.026	0.973
	75	0.458	5.980	0.808	0.030	0.984
	100	0.413	8.284	0.703	0.020	0.790
	125	0.468	9.082	0.829	0.014	0.868
	150	0.456	10.064	0.816	0.023	0.952
1.5 g L^–1^	50	0.169	5.041	0.938	0.011	0.927
	75	0.215	6.946	0.890	0.025	0.989
	100	0.285	7.638	0.805	0.021	0.894
	125	0.381	7.953	0.879	0.016	0.919
	150	0.430	7.997	0.920	0.017	0.964
1.75 g L^–1^	50	0.215	4.371	0.985	0.014	0.964
	75	0.190	6.782	0.991	0.015	0.964
	100	0.223	7.916	0.990	0.019	0.982
	125	0.186	9.472	0.982	0.020	0.975
	150	0.211	10.279	0.975	0.026	0.984

### Isotherm models

The sorption isotherm defines the association between the biosorbent and the measure of analytic substance in the solution. To explain the mechanism of AB 36 and DB86 dyes biosorption to the biochar, the Langmuir model (LGM) and Freundlich (FDM) model were fitted to the experimental data^95–97^.

The LGM assumes that on a homogeneous biosorbent surface, monolayer sorption can take place with no interaction between the sorbates. The linear form of this model is defined by Eq. (12).

12 $$\frac{{{C_e}}}{{{q_e}}}=\left( {\frac{1}{{{q_m}}}} \right){C_e}+\frac{1}{{{q_m}{K_L}}}$$

*q*_m_, *q*e, *C*_*e*_ and *K*_L_ signify the optimum biosorption capacity (mg/g), biosorption capacity at equilibrium (mg/g), sorbate concentration in the solution at equilibrium (mg/L) and the LGM constant (L/mg). The sorption effect of the biosorption process can be described by the equilibrium constant R_L_ of the LGM^98^.

An experimental calculation that is based on the theory that the sorption procedure happens at diverse surfaces having various accessible binding sites with irregular adsorption energies is the FDM. Hence, this model shows that the sorption sites with extreme affinity are filled first. The linear form of this model is given by Eq. (13)^99,100^.

13 $$\log {q_e}=\log {K_F}+\frac{1}{n}\log {C_e}$$

K_F_ and 1/n represent the FDM parameters related to the biosorption capacity and intensity. A favourable biosorption corresponds to a value of 1 < *n* < 10^101,102^.

The Redlich-Peterson isotherm model (RPM) is a blend of the LGM and FDM. The numerator is the LGM and has the advantage of approaching the Henry region at unlimited dilution. This model is an experimental isotherm model that incorporates three factors. It blends elements from the LGM and FDM equation, hence the mechanism of sorption is a blend and does not follow an ideal monolayer sorption. The linearized form of this model is given by Eq. (14)^103^.

14 $$\ln \frac{{{C_e}}}{{{q_e}}}=\beta \ln {C_e} - \ln A$$

$\beta$, and A represents dimensionless RP exponent parameter that lies between 0 and 1 and RPM constant (L/mg)^104^.

**Fig. 12:**
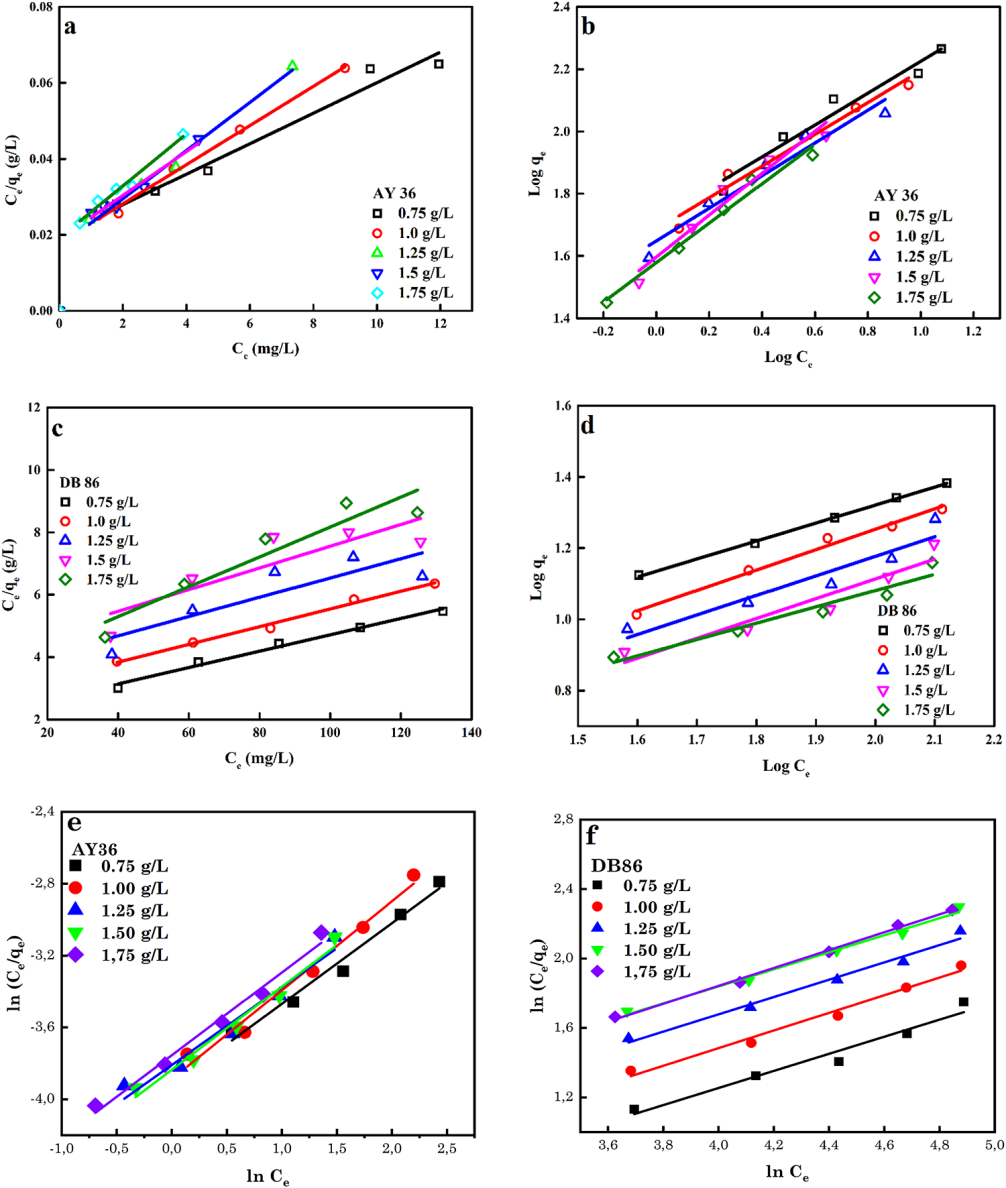
Linear plots of (a and b) LGM and FDM for the biosorption of AB36 dye, (c and d) LGM and FDM for the biosorption of DB86 dye and (e and f) Redlich model for the biosorption of AB36 and DB86 dyes.

The linear plots of all models are given in Fig. 12. The parameters determined by all models in Tables 7 and 8 show that the LGM had higher *R*^2^ values than the FDM and RPM *R*^2^ values, except for RPM R^2^ values for 1.75 g/L dosage for adsorption of DB86 dye. Because the dye molecules were evenly and uniformly distributed over the biosorbent’s porous surface, the LGM provided the best description of the biosorption of both dye molecules to the biosorbent. The determined biosorption capacities for both dyes were 270.27 mg/g (AY36 dye) and 36.23 mg/g (DB86 dye). The biosorption process suggested that both dyes were biosorbed to the biosorbent in a monolayer. When compared to various biosorbents that have been utilized throughout the years for the confiscation of both dyes, it was found from the considered literature summarised in Table 9 that the biosorption of both dyes to the produced biosorbent was outstanding. The relatively lower adsorption capacity for DB86 dye compared to AY36 dye (*q*_m_ values of 36.23 for DB86 and 270.27 for AY36 dye) (Table 9) may be attributed to the molecular structure (reduced dye molecular structures are readily adsorbed into the pores of porous materials), size, and FGs (electron density of the anionic functional and the steric effect of the dye molecules performance a major role in the adsorption rate determination) of the dyes. Variations in these factors may impact how the dye molecules and the DRB-S biosorbent interact^105–107^.

**Table 7:** Deteremined LGM, FDM, and RPM constants and calculated parameters for AY36 dye utilizing different DRB-S dosage.

Isotherm	Isotherm	DRB-S concentrations				
Model	Parameters	0.75 g/L	1 g/L	1.25 g/L	1.5 g/L	1.75 g/L
LGM	*q*_*m*_ (mg/g)	270.27	192.31	144.93	142.86	120.48
	*K*_*L*_ x 10^3^	0.2	0.3	0.5	0.5	0.6
	*R* ^*2*^	0.9967	0.9973	0.9984	0.9989	0.9973
FDM	*K*_*F*_	0.53	0.52	0.50	0.47	0.46
	*n*	1.96	1.96	1.90	1.48	1.58
	*R* ^*2*^	0.9850	0.9810	0.9680	0.9760	0.9990
RPM	$\:\beta\:$	0.45	0.50	0.43	0.46	0.46
	*A*	1.37	1.36	1.34	1.35	1.32
	*R* ^*2*^	0.9775	0.9803	0.9598	0.9776	0.9848

**Table 8:** Determined LGM, FDM, and RPM constants and calculated parameters for DB86 dye using different DRB-S dosage.

Isotherm	Isotherm	DRB-S concentrations				
Model	Parameters	0.75 g/L	1 g/L	1.25 g/L	1.5 g/L	1.75 g/L
LGM	*q*_*m*_ (mg/g)	36.2300	28.0900	23.6400	20.7000	19.5700
	*K*_*L*_ x 10^3^	0.01430	0.0149	0.0143	0.0135	0.0149
	*R* ^*2*^	0.97880	0.9981	0.9924	0.9961	0.9949
FDM	*K*_*F*_	18.300	16.700	15.400	14.500	15.300
	*n*	2.0430	1.7310	1.3710	1.1300	1.232
	*R* ^*2*^	0.9630	0.982	0.9830	0.9910	0.994
RPM	$\:\beta\:$	0.4900	0.5100	0.5000	0.4900	0.5200
	*A*	0.7200	0.5500	0.3200	0.1200	0.2200
	*R* ^*2*^	0.9608	0.9833	0.9830	0.9897	0.9966

**Table 9:** Comparison of the maximum biosorption capacities of DRB-S employed for removing AY36 and DB86 dyes.

Biosorbents	q_m_ (mg/g)	References
DRB-S	270.27 (AY36)	This study
DRB-S	36.23 (DB86)	This study
Cellulose hydrogel	53.76 (DB86)	^108^
Manioc husk	6.1 (DB86)	^109^
Alginate-encapsulated activated carbon	21.6 (DB86)	^110^
N-doping activated carbons from fish waste and sawdust	232.56 (AY36)	^80^

### Adsorption mechanism of MB dye by DRB-S

**Fig. 13:**
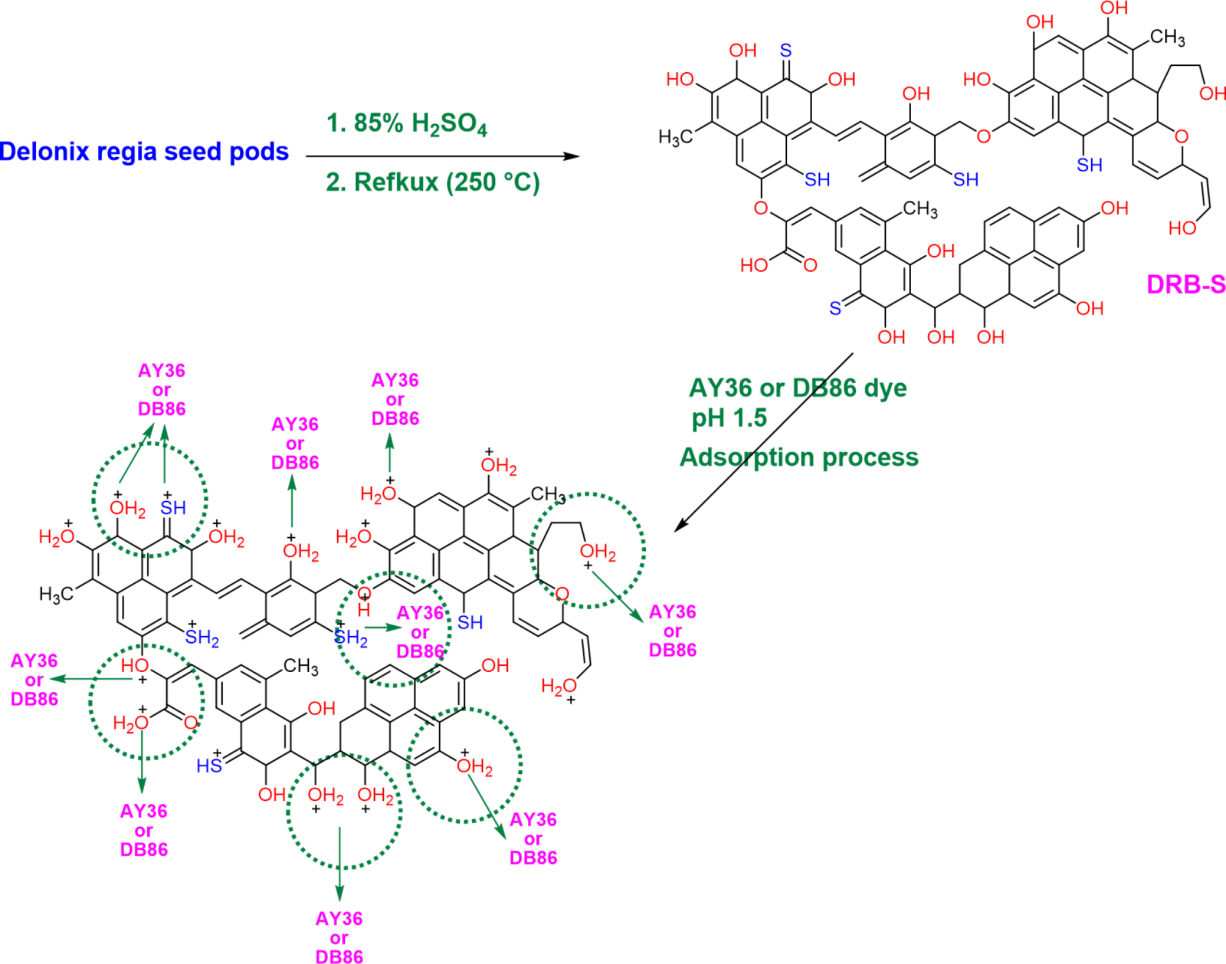
The likely mechanism by which the AY36 and DB86 dyes are adsorbed onto the DRB-S.

Figure 13 explains the likely mechanism by which DRB-S absorbed the AY36 dye and DB86 dye ions. Following the 85% H_2_SO_4_ dehydration of the DPSPs (Delonix regia raw material). According to FTIR analysis, various FGs, including C=O, COOH, C–O–C, hydroxyl O–H, C–S, and SH groups, developed on the surface of the adsorbent (DRB-S). Because of the electrostatic interaction between the oxygen lone pair on the DRB-S surface and the positive charge on the sulphur atom of the AY36 dye and DB86 dye, the adsorption mechanism of the AY36 dye and DB86 dye ions in an acidic medium (pH 1.5) can be accomplished through physical interaction. Once the surface charge became positive, the acidic pH of the acidic medium attracted ions.

In an acidic environment, the surface of biochar picks up a positive charge, which attracts negatively charged dye molecules^111–113^. Additionally, the negative ions in the solution interact with the FGs of positive ions on the surface of the DRB-S. Additionally, dye molecules are more soluble at an acidic pH, facilitating their diffusion through the pores in the DRB-S and their attachment to the adsorption sites. Biochar-S is an excellent method for removing color from industrial effluent because the acidic pH is essential for encouraging the adsorption of AY36 dye and DB86 dye molecules onto the material. The most significant process is the adsorption of ionizable organic molecules to the positively charged surface of the biochar via electrostatic interaction^113^. How successfully an aqueous solution attracts or repels impurities depends on its pH and ionic strength^113,114^.

Furthermore, the pH of the solution influences the capacity of organic contaminants in industrial effluent to adsorb^115^. Parshetti et al.‘s study^116^ examined the use of food waste-derived biochar in the adsorption of textile colours in wastewater. They found that an alkaline pH enhanced the adsorption of dyes. The significant interaction between the negatively charged sites on the biochar surface and the positively charged dyes explained it^117^. However, since there was an excess of H^+^ at pH 1.5, which competed with the positive charges of the dye, it was less successful at adsorbing organic dye^116^. Tsai and Chen^117^ and Xu et al.^118^ have noted that pH impacts biochar’s capacity to absorb materials. As a result, the charged sites are altered by the pH of the solution, which alters the ability of organic and inorganic contaminants from industrial effluent to adsorb on biochar^119–122^. The hydrogen bonding among hydrogen donating OH groups on the DRB-S surface and nitrogen or oxygen atoms in the dyes (hdrogen acceptors) are termed dipole-dipole hydrogen bonding. Another probalbe hydrogen bonding interaction is the Yoshide bonding which exists between the aromatic rings present in the dyes and the OH groups that exist on the surface of the DRB-S adsorbent. Also, the existence of electron accepting aromatic rings on the dyes and electron donating oxygen groups on the biosorbent surface may give rise to the n-π interaction. The adsorption mechanism can also explained by π -π interaction which involved the intermolecular force of attraction between the organic molecules containing benzene rings or thr C=C bonds^123,124^.

### Regeneration study

**Fig. 14:**
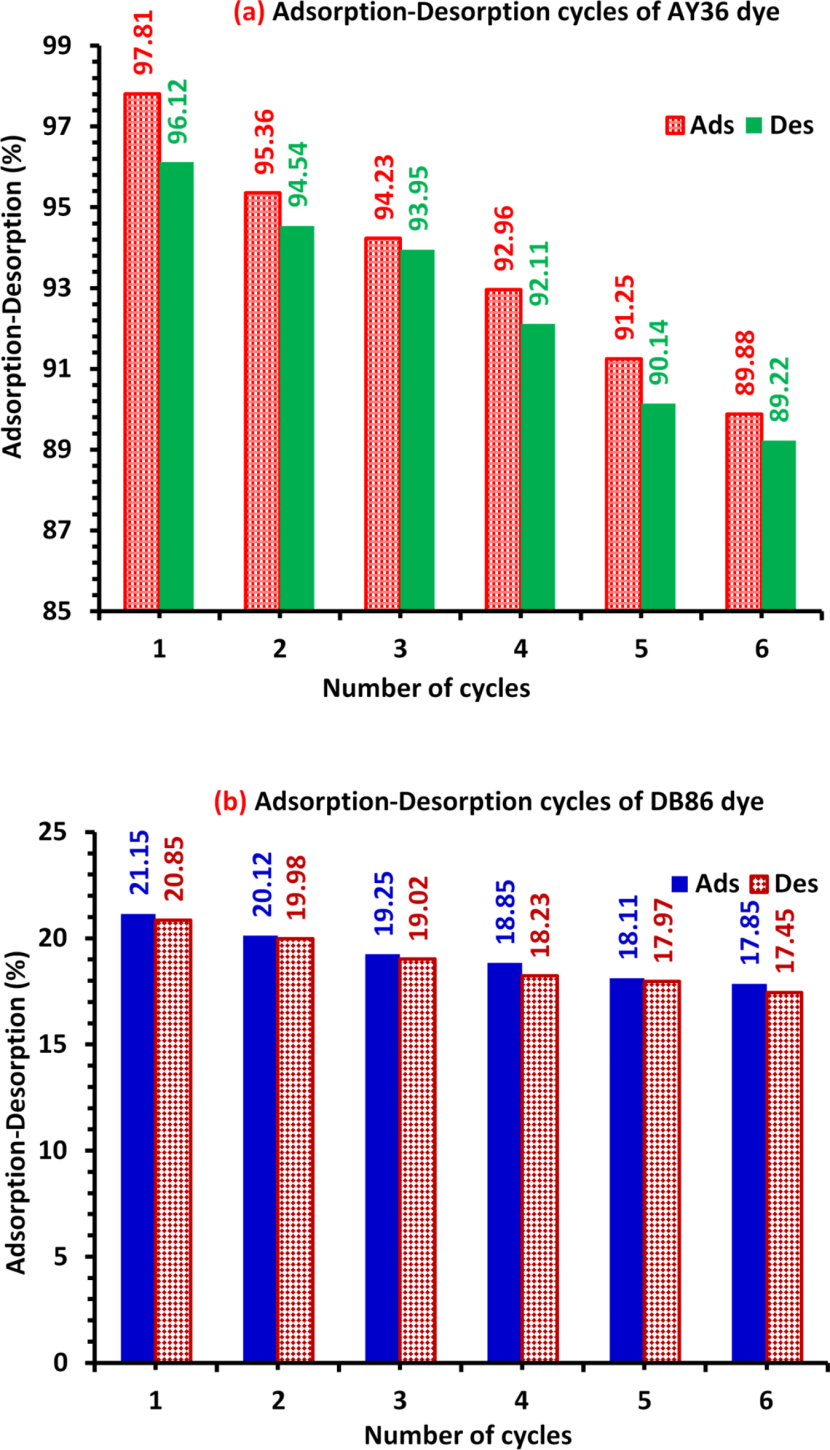
Renewal study of (a) AY36 dye and (b) DB86 dye adsorption-desorption by DRB-S adsorbent using dye *C*_0_ (100 ppm) and 1.0 g/L DRB-S dose at room temperature.

Much research has demonstrated that regeneration processes, including thermal, microwave irradiation, moist air oxidation, microbial/biological, chemical/solvent, and others, are energy-efficient and can use little money, according to a recent study by Aragaw and Bogale^125^. NaOH and HCl were found to be among the best activating agents, exhibiting superior performance throughout the desorption process. It has been observed that chemical/solvent regeneration is employed more frequently when the adsorbents have many efficient adsorption-desorption cycles^125^. Desorption tests were conducted on the AY36 and DB86 dyes from the DRB-S adsorbent using 0.1 M NaOH as an elution desorption media. The concentrations of the dyes were measured, and the DRB-S was then reactivated using 0.1 M HCl. This was done to examine the viability and adsorbent reusability of the adsorption of AY36 and DB86 dyes. The percentage of dye desorption in this work reduced as the regeneration cycles increased (Fig. 14a). Six adsorption/desorption cycles have been examined using the regenerated DRB-S. The variations in adsorption and desorption were consistent across the cycles^40,41,43^. Nevertheless, after six cycles, it dropped by around 3.3% for DB86 dye and 8.59% for AY36 dye. DRB-S might be applied as a long-lasting water dye removal method for AY36 and DB86 dyes (Fig. 14b).

## Conclusion

The present study aims to eliminate two hazardous dyes (AY36 and DB86 dyes) from the water cycle using DRB-S. The optimal pH for AY36 dye and DB86 dye adsorption to the DRB-S adsorvbent was at pH 1.5. For the adsorption of AY36 and DB86 to DRB-S, equilibrium was attained at 30 and 90 min of reaction time interaction. The LGM and PSOM were found to excellently define the biosorption of both dye molecules to the biosorbent. The determined biosorption capacities for both dyes (AY36 and DB86) were 270.27 mg/g and 36.23 mg/g, respectively. Accordingly, this novel synthesized DRB-S adsorbent had an outstanding sorption capacity. It effectively removed AY36 and DB86 dyes, signifying their potential utilization for wastewater treatment and that they can be reused without any loss to their adsorption efficiency. To enhance the study’s findings, it is recommended to further optimize adsorption conditions, especially for DB86 dye. Additionally, testing the adsorbent in real wastewater conditions and conducting pilot-scale studies would help evaluate its practical application. Lastly, examining the environmental impact and potential toxicity of the dye-laden adsorbent is crucial to ensure the safety and sustainability of the biosorption process for wastewater treatment.

## Electronic supplementary material

Below is the link to the electronic supplementary material.

Supplementary Material 1

## Data Availability

Data will be available upon request from the corresponding author.
