# Supplementary material for: Adsorption of Acid Yellow 36 and direct blue 86 dyes to Delonix regia biochar-sulphur
Source: Sci Rep. 2025 Jan 27;15:3448. doi: 10.1038/s41598-025-85405-4 (PMC11772611; doi:10.1038/s41598-025-85405-4)
Supplement: Supplementary file 1 — Supplementary Material 1 [file 41598_2025_85405_MOESM1_ESM.docx]

**Supplementary Data**

**Adsorption of Acid Yellow 36 and Direct Blue 86 Dyes to *Delonix Regia* Biochar-Sulphur**

Ahmed Eleryan^1^, Uyiosa Osagie Aigbe^2^, Kingsley Eghonghon Ukhurebor^3^, Mohamed A. Hassaan^1^, Safaa Ragab^1^, Otolorin Adelaja Osibote^2^, Ismail Hossain^4^, Ahmed El Nemr^1*^

^1^ National Institute of Oceanography and Fisheries (NIOF), Kayet Bey, Elanfoushy, Alexandria, Egypt

^2^ Department of Mathematics and Physics, Cape Peninsula University of Technology, Cape Town, South Africa

^3^ Department of Physics, Edo State University, Uzairue, Edo State, Nigeria

^4^Department of Nuclear and Renewable Energy, Ural Federal University, Yekaterinburg, Russia

[aeleryan71@yahoo.com](mailto:aeleryan71@yahoo.com) (A. Eleryan), [uyi4we@gmail.com](mailto:uyi4we@gmail.com) (U.O. Aigbe), [ukeghonghon@gmail.com](mailto:ukeghonghon@gmail.com) (K.E. Ukhurebor), [hossain.ismail44@yahoo.com](mailto:hossain.ismail44@yahoo.com) (I. Hossain), [safaa_ragab65@yahoo.com](mailto:safaa_ragab65@yahoo.com) (S. Ragab), [osibotea@cput.ac.za](mailto:osibotea@cput.ac.za) (O.A. Osibote), [mhss95@mail.com](mailto:mhss95@mail.com) (M.A. Hassaan),

^*^Corresponding Author: E-mail: [ahmedmoustafaelnemr@yahoo.com](mailto:ahmedmoustafaelnemr@yahoo.com); [ahmed.m.elnemr@gmail.com](mailto:ahmed.m.elnemr@gmail.com) (A. El Nemr)

Table S1. Effect of DRB-S dose (g/L) on the removal % of different initial AY36 dye at different time (15-150 min).

| DRB-S |  | 50 ppm | 75 ppm | 100 ppm | 125 ppm | 150 ppm |
| --- | --- | --- | --- | --- | --- | --- |
| g/L | time-min | % removal of AY36 dye | | | | |
| 0.75 | 15 | 85.46 | 84.45 | 79.99 | 76.51 | 75.10 |
|  | 30 | 92.51 | 90.69 | 87.55 | 79.44 | 80.28 |
|  | 45 | 92.66 | 92.23 | 89.85 | 81.92 | 81.57 |
|  | 60 | 93.23 | 93.19 | 91.65 | 85.49 | 84.65 |
|  | 90 | 94.53 | 95.39 | 92.08 | 87.85 | 88.00 |
|  | 120 | 95.68 | 95.87 | 95.03 | 91.31 | 91.75 |
|  | 150 | 96.55 | 95.97 | 95.25 | 93.61 | 92.42 |
| 1.00 | 15 | 89.06 | 91.56 | 85.89 | 85.95 | 79.42 |
|  | 30 | 92.95 | 93.57 | 89.92 | 89.92 | 85.89 |
|  | 45 | 93.67 | 94.63 | 91.79 | 92.57 | 90.40 |
|  | 60 | 94.24 | 95.30 | 93.16 | 93.26 | 91.84 |
|  | 90 | 95.25 | 96.35 | 93.88 | 94.47 | 92.66 |
|  | 120 | 96.40 | 96.93 | 95.97 | 95.22 | 93.09 |
|  | 150 | 97.70 | 97.41 | 96.40 | 95.45 | 94.00 |
| 1.25 | 15 | 89.78 | 93.09 | 89.35 | 87.27 | 83.45 |
|  | 30 | 93.95 | 94.72 | 92.15 | 92.63 | 88.92 |
|  | 45 | 94.53 | 95.59 | 94.39 | 92.86 | 91.03 |
|  | 60 | 94.96 | 95.97 | 95.32 | 95.22 | 93.86 |
|  | 90 | 95.97 | 96.45 | 95.83 | 95.74 | 94.67 |
|  | 120 | 96.83 | 97.12 | 96.98 | 96.31 | 94.96 |
|  | 150 | 98.27 | 97.89 | 97.41 | 96.95 | 95.11 |
| 1.50 | 15 | 91.08 | 93.95 | 91.22 | 91.36 | 86.80 |
|  | 30 | 94.67 | 95.59 | 93.74 | 94.70 | 92.85 |
|  | 45 | 95.11 | 95.78 | 95.32 | 95.51 | 94.48 |
|  | 60 | 95.68 | 96.16 | 95.97 | 96.55 | 94.96 |
|  | 90 | 96.40 | 96.93 | 96.47 | 96.89 | 95.59 |
|  | 120 | 97.12 | 97.50 | 97.41 | 97.29 | 96.16 |
|  | 150 | 98.70 | 98.56 | 98.27 | 97.87 | 97.07 |
| 1.75 | 15 | 91.08 | 93.95 | 91.22 | 91.36 | 86.80 |
|  | 30 | 94.67 | 95.59 | 93.74 | 94.70 | 92.85 |
|  | 45 | 95.11 | 95.78 | 95.32 | 95.51 | 94.48 |
|  | 60 | 95.68 | 96.16 | 95.97 | 96.55 | 94.96 |
|  | 90 | 96.40 | 96.93 | 96.47 | 96.89 | 95.59 |
|  | 120 | 97.12 | 97.50 | 97.41 | 97.29 | 96.16 |
|  | 150 | 98.70 | 98.56 | 98.27 | 97.87 | 97.07 |

Table S2. Effect of DRB-S dose (g/L) on the removal % of different initial DB86 dye at different time (15-150 min).

| DRB-S |  | 50 ppm | 75 ppm | 100 ppm | 125 ppm | 150 ppm |
| --- | --- | --- | --- | --- | --- | --- |
| g/L | time-min | % removal of DB86 dye | | | | |
| 0.75 | 15 | 5.97 | 4.58 | 4.20 | 3.47 | 3.05 |
|  | 30 | 12.42 | 7.21 | 6.28 | 5.12 | 4.86 |
|  | 45 | 13.46 | 8.60 | 7.94 | 6.38 | 5.06 |
|  | 60 | 14.08 | 11.79 | 9.19 | 7.21 | 6.45 |
|  | 90 | 19.07 | 13.87 | 13.25 | 10.21 | 7.84 |
|  | 120 | 19.70 | 14.56 | 14.18 | 12.37 | 9.29 |
|  | 150 | 19.96 | 16.33 | 14.48 | 13.16 | 12.07 |
| 1.00 | 15 | 9.29 | 8.74 | 7.94 | 6.88 | 5.27 |
|  | 30 | 12.62 | 9.43 | 8.15 | 7.46 | 6.24 |
|  | 45 | 14.29 | 13.32 | 12.00 | 9.46 | 8.25 |
|  | 60 | 17.20 | 14.43 | 13.46 | 11.21 | 9.23 |
|  | 90 | 19.70 | 15.12 | 13.77 | 12.96 | 10.61 |
|  | 120 | 19.90 | 16.23 | 14.08 | 14.45 | 10.82 |
|  | 150 | 20.60 | 18.31 | 16.89 | 14.60 | 13.59 |
| 1.25 | 15 | 13.66 | 11.10 | 10.65 | 9.79 | 8.95 |
|  | 30 | 16.37 | 13.32 | 12.73 | 11.62 | 9.71 |
|  | 45 | 18.45 | 16.37 | 15.02 | 13.46 | 11.51 |
|  | 60 | 19.07 | 16.92 | 15.22 | 13.62 | 11.86 |
|  | 90 | 19.90 | 17.62 | 15.43 | 13.87 | 12.14 |
|  | 120 | 20.74 | 18.03 | 15.54 | 14.12 | 12.48 |
|  | 150 | 23.44 | 18.53 | 15.68 | 14.79 | 15.95 |
| 1.50 | 15 | 16.58 | 14.84 | 12.10 | 10.46 | 9.99 |
|  | 30 | 17.41 | 15.67 | 13.66 | 11.62 | 10.61 |
|  | 45 | 18.45 | 17.20 | 15.12 | 13.21 | 11.58 |
|  | 60 | 19.28 | 17.75 | 15.43 | 13.54 | 12.42 |
|  | 90 | 19.90 | 18.17 | 15.64 | 13.87 | 12.76 |
|  | 120 | 21.15 | 18.45 | 15.85 | 14.20 | 13.18 |
|  | 150 | 24.27 | 18.70 | 16.04 | 15.79 | 16.30 |
| 1.75 | 15 | 18.24 | 17.75 | 15.22 | 14.12 | 13.18 |
|  | 30 | 19.28 | 18.31 | 15.74 | 14.54 | 13.52 |
|  | 45 | 20.53 | 18.73 | 16.37 | 14.95 | 13.94 |
|  | 60 | 21.78 | 19.42 | 16.99 | 15.29 | 14.36 |
|  | 90 | 22.61 | 19.84 | 17.62 | 15.79 | 14.84 |
|  | 120 | 23.86 | 20.67 | 18.14 | 16.12 | 15.26 |
|  | 150 | 27.39 | 21.64 | 18.34 | 16.37 | 16.85 |

Table S3. Effect of the DRB-S dose and initial AY36 dye concentration on the *C*_e_ (ppm) and *q*_e_ (mg/g).

| DRB-S | *C*_0_ (ppm) | *C*_e_ (ppm) | *q*_e_ (mg/g) |
| --- | --- | --- | --- |
| 0.75 g/L | 50 | 1.73 | 64.36 |
|  | 75 | 3.02 | 95.97 |
|  | 100 | 4.75 | 127.00 |
|  | 125 | 7.99 | 156.02 |
|  | 150 | 11.37 | 184.84 |
| 1.0 g/L | 50 | 1.15 | 48.85 |
|  | 75 | 1.94 | 73.06 |
|  | 100 | 3.60 | 96.40 |
|  | 125 | 5.69 | 119.31 |
|  | 150 | 9.00 | 141.00 |
| 1.25 g/L | 50 | 0.86 | 39.31 |
|  | 75 | 1.58 | 58.73 |
|  | 100 | 2.59 | 77.93 |
|  | 125 | 3.81 | 96.95 |
|  | 150 | 7.34 | 114.13 |
| 1.5 g/L | 50 | 0.65 | 32.90 |
|  | 75 | 1.08 | 49.28 |
|  | 100 | 1.73 | 65.52 |
|  | 125 | 2.66 | 81.56 |
|  | 150 | 4.39 | 97.07 |
| 1.75 g/L | 50 | 0.50 | 28.28 |
|  | 75 | 0.94 | 42.32 |
|  | 100 | 1.58 | 56.24 |
|  | 125 | 2.30 | 70.11 |
|  | 150 | 3.89 | 83.94 |

Table S4. Effect of the DRB-S dose and initial DB86 dye concentration on the *C*_e_ (ppm) and *q*_e_ (mg/g).

| DRB-S | *C*_0_ (ppm) | *C*_e_ (ppm) | *q*_e_ (mg/g) |
| --- | --- | --- | --- |
| 0.75 g/L | 50 | 40.02 | 13.30 |
|  | 75 | 62.75 | 16.34 |
|  | 100 | 85.52 | 19.31 |
|  | 125 | 108.55 | 21.93 |
|  | 150 | 131.90 | 24.14 |
| 1.0 g/L | 50 | 39.70 | 10.30 |
|  | 75 | 61.27 | 13.73 |
|  | 100 | 83.11 | 16.89 |
|  | 125 | 106.74 | 18.26 |
|  | 150 | 129.61 | 20.39 |
| 1.25 g/L | 50 | 38.28 | 9.38 |
|  | 75 | 61.10 | 11.12 |
|  | 100 | 84.32 | 12.54 |
|  | 125 | 106.52 | 14.79 |
|  | 150 | 126.07 | 19.14 |
| 1.5 g/L | 50 | 37.86 | 8.09 |
|  | 75 | 60.98 | 9.35 |
|  | 100 | 83.96 | 10.69 |
|  | 125 | 105.27 | 13.16 |
|  | 150 | 125.55 | 16.30 |
| 1.75 g/L | 50 | 36.30 | 7.83 |
|  | 75 | 58.77 | 9.27 |
|  | 100 | 81.66 | 10.48 |
|  | 125 | 104.54 | 11.69 |
|  | 150 | 124.72 | 14.45 |
